# PAC_1_, VPAC_1_, and VPAC_2_ Receptor Expression in Rat and Human Trigeminal Ganglia: Characterization of PACAP-Responsive Receptor Antibodies

**DOI:** 10.3390/ijms232213797

**Published:** 2022-11-09

**Authors:** Zoe Tasma, Andrew Siow, Paul W. R. Harris, Margaret A. Brimble, Simon J. O’Carroll, Debbie L. Hay, Christopher S. Walker

**Affiliations:** 1 School of Biological Sciences, The University of Auckland, Auckland 1010, New Zealand; 2 School of Chemical Sciences, The University of Auckland, Auckland 1010, New Zealand; 3 Maurice Wilkins Centre for Molecular Biodiscovery, The University of Auckland, Auckland 1010, New Zealand; 4 Department of Anatomy and Medical Imaging, and Centre for Brain Research, Faculty of Medical and Health Science, The University of Auckland, Auckland 1023, New Zealand; 5 Department of Pharmacology and Toxicology, The University of Otago, Dunedin 9016, New Zealand

**Keywords:** PACAP, VIP, PAC_1_ receptor, VPAC_1_ receptor, VPAC_2_ receptor, migraine, trigeminal ganglia

## Abstract

Pituitary adenylate cyclase-activating peptide (PACAP) is a neuropeptide expressed in the trigeminal ganglia (TG). The TG conducts nociceptive signals in the head and may play roles in migraine. PACAP infusion provokes headaches in healthy individuals and migraine-like attacks in patients; however, it is not clear whether targeting this system could be therapeutically efficacious. To effectively target the PACAP system, an understanding of PACAP receptor distribution is required. Therefore, this study aimed to characterize commercially available antibodies and use these to detect PACAP-responsive receptors in the TG. Antibodies were initially validated in receptor transfected cell models and then used to explore receptor expression in rat and human TG. Antibodies were identified that could detect PACAP-responsive receptors, including the first antibody to differentiate between the PAC_1n_ and PAC_1s_ receptor splice variants. PAC_1_, VPAC_1_, and VPAC_2_ receptor-like immunoreactivity were observed in subpopulations of both neuronal and glial-like cells in the TG. In this study, PAC_1_, VPAC_1_, and VPAC_2_ receptors were detected in the TG, suggesting they are all potential targets to treat migraine. These antibodies may be useful tools to help elucidate PACAP-responsive receptor expression in tissues. However, most antibodies exhibited limitations, requiring the use of multiple methodologies and the careful inclusion of controls.

## 1. Introduction

Expression of the related neuropeptides pituitary adenylate cyclase-activating peptide (PACAP) and vasoactive intestinal peptide (VIP) is well documented in several migraine-relevant structures, such as the trigeminal ganglia (TG), brainstem, cranial vasculature, and other cranial ganglia [1,2,3,4,5,6]. Increasing evidence suggests these peptides may be involved in migraine and headache pathophysiology [7,8,9,10]. For example, studies have shown elevated PACAP concentrations during spontaneous migraine attacks, lower interictal levels of PACAP in migraineurs, and PACAP infusion can induce migraine-like attacks in migraine patients or a mild vascular headache in healthy individuals [7,8,11,12]. The role of VIP in headaches is less well-defined. VIP has been shown to induce headaches, and sustained VIP infusion can induce delayed migraine-like symptoms in migraine patients [9,10,13]. The underlying pathogenic mechanisms used by PACAP and VIP to induce migraine-like attacks are unclear. It is possible that PACAP and VIP could activate overlapping or discrete mechanisms. Presumably, both peptides directly or indirectly activate TG sensory neurons, which are essential for conducting nociceptive signals in the head. However, the precise site of action and the specific receptor involved is not known for either peptide.

PACAP and VIP display activities at three distinct G protein-coupled receptors (GPCRs). PACAP can activate the PAC_1_, VPAC_1_, and VPAC_2_ receptors, whereas the actions of VIP are usually attributed to the VPAC_1_ or VPAC_2_ receptors [14,15]. The PAC_1_ receptor can generate many additional variants due to alternative splicing [16]. PAC_1n_ is considered the reference receptor as it has no deletions or insertions. N-terminal deletion variants can exhibit differential agonist profiles, as shown for the PAC_1s_ receptor where VIP is a potent agonist, and intracellular loop 3 (ICL3) insertion variants are predicted to influence receptor regulation [15,17]. Monoclonal antibodies against PACAP and one of its receptors, PAC_1_, have been developed for the treatment of migraine [18]. Evaluation of the anti-PACAP antibody efficacy is ongoing, but the anti-PAC_1_ receptor antibody exhibited no significant clinical efficacy over placebo for the treatment of migraine, resulting in uncertainty around the role PAC_1_ plays in migraine [19]. This may indicate that the trialed anti-PAC_1_ antibody does not have sufficient target engagement to reduce PAC_1_ activation or that the blockade of PAC_1_ alone is insufficient to treat migraine. However, it is also possible that PACAP contributes to migraine through VPAC_1_, VPAC_2_, or another PAC_1_ splice variant. There is currently no clinical data available describing the effects of blocking VIP, VPAC_1_, or VPAC_2_ activity in migraine.

Data describing PAC_1_, VPAC_1_, and VPAC_2_ receptor expression in the TG is primarily mRNA [2,6,20,21]. These studies indicate that the PAC_1_ receptor, including the PAC_1n_ and PAC_1s_ splice variants, is the predominant subtype present, with relatively low levels of VPAC_1_ and VPAC_2_ mRNA. mRNA data is useful as a guide for examining receptor expression. However, mRNA may not correlate with protein expression as translation may not be equivalent in all tissues, or the translated protein may be transported to a spatially different location [22]. Therefore, determining the location of protein expression is necessary.

Antibodies are important tools for examining protein expression. However, the data associated with them is becoming increasingly scrutinized. Many antibodies are not well validated and exhibit poor specificity, which contributes to a lack of reproducibility [23,24]. To avoid these issues and ensure published datasets are robust, several antibody validation guidelines have been developed, in which all recommend the inclusion of a genetic approach [25,26,27]. GPCRs are particularly complicated as they typically exhibit relatively low protein expression and, therefore, require antibodies with high specificity and sensitivity [28,29]. Furthermore, GPCRs exist in multiple conformations which can complicate targeting an immunogenic region with high-affinity and many GPCRs exist as splice variants whose considerable homology may lead to selectivity issues [30,31,32].

PAC_1_, VPAC_1_, and VPAC_2_ receptor protein expression has been investigated using antibodies. Prior validation of anti-PACAP-responsive receptor antibodies has relied on Western blotting, while genetic approaches, such as knockout models or transfected cells, are rarely employed [5,33,34,35]. Although useful, Western blotting may not translate to other applications such as immunofluorescence and IHC, as the target epitope could be masked or be conformationally altered [24,26,36,37]. These issues are evident for PAC_1_ receptor expression where different studies have reported either the presence or absence of PAC_1_ in the cell bodies of TG neurons [33,38,39,40,41]. This discrepancy has been reported between studies using the same antibody, published by the same research group [33,41]. Overall published results for PAC_1_ are difficult to interpret, many of the antibodies used are no longer available and are not well-validated, and none have looked at splice variant protein expression [5,33,35,38,39,42]. In contrast to PAC_1_, relatively few studies have examined the protein expression of VPAC_1_ and VPAC_2_ in the TG. Despite very low reported mRNA levels, protein was detected which emphasizes the importance of examining protein rather than mRNA for an accurate description of expression [33,40,43].

To better understand the role PACAP-responsive receptors, especially the PAC_1_ receptor, play in migraine and headache disorders and realize the therapeutic benefit of targeting this receptor family, a more comprehensive understanding of their expression is required. The lack of well-validated anti-receptor antibodies currently limits this. Therefore, this study characterized commercially available antibodies targeting the PAC_1_, VPAC_1_, and VPAC_2_ receptors using immunofluorescence and Western blotting in a receptor transfected cell model, representing a genetic approach [26,27]. A subset of antibodies was then used to examine receptor expression in rat and human TG.

## 2. Results

### 2.1. Characterization of Anti-PAC_1_ Receptor Antibodies

**Figure 1:**
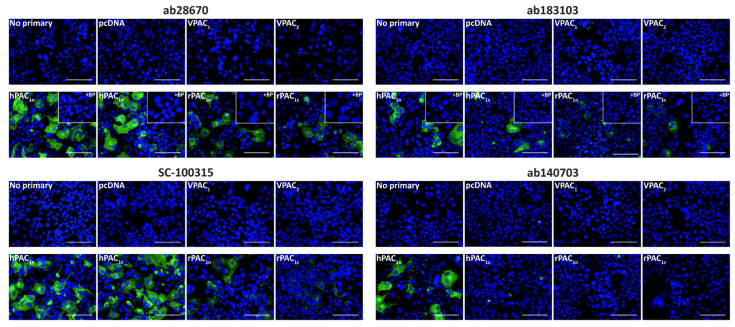
PAC_1_ receptor-like immunoreactivity detected in transfected Cos7 cells. Cells were transfected with the hPAC_1n_, hPAC_1s_, rPAC_1n_, rPAC_1s_, hVPAC_1_ and hVPAC_2_ receptors or vector (pcDNA3.1^+^). Cells were stained with ab28670 (1:1000) or subjected to antigen retrieval before addition of ab183103 (1:100), SC-100315 (1:50) or ab140703 (1:100). Pre-absorbed blocking peptide (BP) antibody controls for certain antibodies are presented as insets. Antibody immunoreactivity is shown in green and nuclear DAPI staining in blue. Images were taken with a 40× non-confocal objective and represent one field of view from three independent experiments. Scale bar, 100 µm.

The specificity of anti-PAC_1_ receptor antibodies was evaluated using fluorescent ICC and Western blotting in transfected Cos7 cells. Cos7 cells were chosen as they are reported not to endogenously express a functional PACAP-responsive receptor and therefore qualify as a genetic knockout background for PAC_1_, VPAC_1_, and VPAC_2_ [15]. To confirm robust receptor expression could be detected in these cells, expression of an HA-tagged human PAC_1_ (HA-PAC_1_) receptor was assessed using an anti-HA antibody (Figure S1A). Subsequent immunofluorescence was then conducted using un-tagged PAC_1_ to ensure the HA-tag did not influence staining. All anti-PAC_1_ receptor antibodies exhibited no immunoreactivity in vector, VPAC_1_, or VPAC_2_ receptor transfected cells (Figure 1). The ab28670, ab183103, and SC-100315 antibodies detected immunoreactivity in cells transfected with the human and rat PAC_1n_ and PAC_1s_ receptors (Figure 1). Incubation of ab28670 and ab183103 with their blocking peptides (BPs) resulted in no detectable immunoreactivity, indicating they detect the antigenic region they were raised against. Interestingly, the ab140703 antibody detected only the human PAC_1n_ receptor (Figure 1).

To further validate the anti-PAC_1_ receptor antibodies, Western blotting was performed using protein from transfected Cos7 cells. As with fluorescent ICC, conditions for Western blotting were first tested by detecting HA-PAC_1_ with an anti-HA antibody (Figure S1B,C). Under the Western blotting conditions used in this study, the Benchmark ladder appeared to run at higher molecular weights (MWs) than other protein ladders (Figure S2). Therefore, the apparent MWs observed in blots using this ladder were derived from comparing the Benchmark and Abcam ladders.

**Figure 2:**
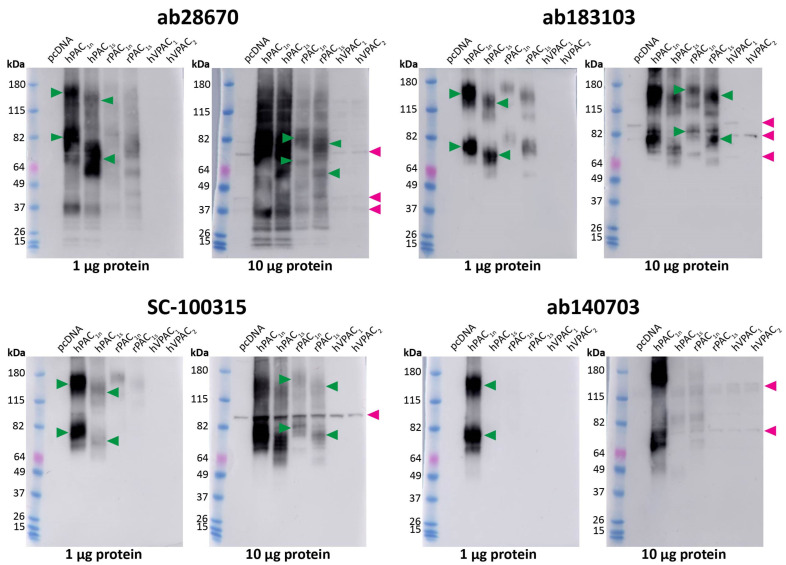
Apparent PAC_1_ receptor molecular weights detected by anti-PAC_1_ receptor antibodies in transfected Cos7 cell protein. Protein samples were prepared from Cos7 cells transfected with the hPAC_1n_, hPAC_1s_, rPAC_1n_, rPAC_1s_, hVPAC_1_ and hVPAC_2_ receptors or vector (pcDNA3.1^+^). Protein was loaded, and blots probed with anti-PAC_1_ receptor antibody (1:1000). The exposure times were 7 s (1 µg) and 2 s (10 µg) for ab28670, 2.5 min (1 µg) and 31 s (10 µg) for ab183103, 15 s (1 µg) and 4 s (10 µg) for SC-100315, 28 s (1 µg) and 14 s (10 µg) for ab140703. Blots are representative of three (1 µg) or two (10 µg) independent experiments. Green arrowheads represent bands of an apparent MW consistent with the anti-HA PAC_1_ control (Figure S1C). Rat PAC_1_ receptor MWs were not clearly observed when 1 µg protein was loaded and are indicated on the 10 µg blot instead. A second green arrowhead indicates a receptor dimer. Pink arrowheads indicate major non-specific bands observed with 10 µg protein.

We then proceeded to test the native receptor antibodies at untagged receptors. Two different amounts of protein (1 μg and 10 μg) were loaded to ensure both specific and non-specific bands could be identified. The ab28670, ab183103, and SC-100315 antibodies detected two immunoreactive smears in human and rat PAC_1_ protein (Figure 2). These bands, described in Table S1, were expected based on the MWs observed for the HA-PAC_1_ control and represent the monomeric and predicted dimeric receptor forms (Figure S1C). However, ab28670 detected several additional bands. Interestingly, a small difference in MW was observed between the PAC_1n_ and PAC_1s_ receptor bands. In contrast, ab140703 detected two immunoreactive smears in only the human PAC_1n_ receptor protein (Figure 2, Table 1). Additional weaker bands were observed with 10 μg protein; however, only the ~50 kDa band in rPAC_1n_ was consistent with the expected receptor size. Excluding ab140703, anti-PAC_1_ receptor antibodies detected immunoreactive bands in lanes loaded with 10 μg but not 1 μg protein from vector transfected cells (Figure 2, Table 1). Several of these bands likely represent the non-specific immunoreactivity of proteins in Cos7 cells as they were also detected in VPAC_1_ and VPAC_2_ receptor protein preparations (Figure 2).

### 2.2. PAC_1_ Receptor-like Immunoreactivity in Rat and Human TG

To evaluate whether antibodies could detect endogenous PAC_1_ receptors histologically, staining with a subset of antibodies was examined in the TG. Images of the independent experiments for rat and human cases are included as supplemental information (Figures S3–S5 and S7). The SC-100315 antibody was not used for immunofluorescent experiments in tissues due to the intensity of the non-specific band at ~68 kDa (Figure 2).

**Figure 3:**
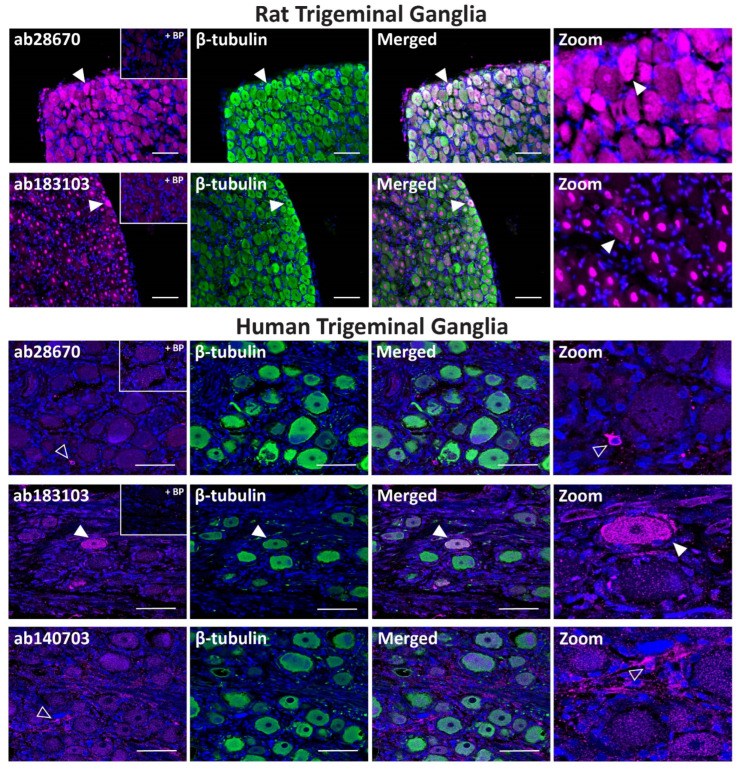
PAC_1_ receptor-like immunoreactivity detected in rat and human trigeminal ganglia. PAC_1_ (ab28670, 1:500; ab183103, ab140703, 1:100) receptor-like immunoreactivity in TG neurons and glia colocalized with β-tubulin (1:500). Pre-absorbed blocking peptide (BP) antibody controls are presented as insets. PAC_1_ antibody immunoreactivity is shown in pink, β-tubulin in green, colocalized regions appear white, and DAPI (rat) or Hoechst (human) stained nuclei in blue. Solid arrowheads indicate examples of immunoreactive neurons and empty arrowheads examples of immunoreactive glia. Images were taken with a 20× non-confocal objective and are representative of TG staining in three individual rat or human cases. Scale bar, 100 µm.

In rat TG, the ab28670 antibody displayed intense PAC_1_ receptor-like immunoreactivity in many neuronal cell bodies that colocalized with the neuronal marker β-tubulin (Figure 3, Table 2). In contrast, the ab183103 antibody exhibited intense immunoreactivity in neuronal nuclei (Figure 3, Table 2). Less pronounced neuronal cell body staining was occasionally observed, which rarely colocalized with β-tubulin. The preincubation of ab28670 and ab183103 with their BPs greatly reduced neuronal and nuclear staining (Figure 3).

In human TG, the ab28670 antibody exhibited no detectable immunoreactivity in neuronal cell bodies but stained occasional glial-like cells, which was abolished in the presence of BP (Figure 3, Table 2). In contrast, the ab183103 antibody stained the occasional neuronal cell body (Figure 3, Table 2). Initial characterization indicated that the ab140703 antibody could detect human PAC_1_ receptor protein and was included for human TG experiments. ab140703 did not exhibit strong immunoreactivity in human TG with variable staining between cases (Figure 3, Table 2). In the presented image, immunoreactive glial-like cells were detected, and more convincing neuronal staining was observed in independent experiments (Figure S4).

### 2.3. Evaluation of PAC_1_ Receptor Antibodies with Western Blotting in Rat and Human TG

**Figure 4:**
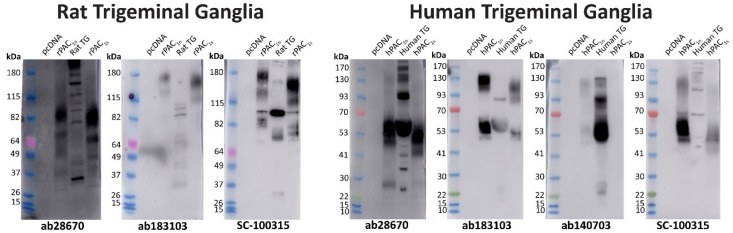
PAC_1_ receptor apparent molecular weights detected in rat and human trigeminal ganglia. Protein samples were prepared from TG or Cos7 cells transfected with the human or rat PAC_1n_ and PAC_1s_ receptors or vector (pcDNA3.1^+^_)_. Protein was loaded, and blots probed with anti-PAC_1_ receptor antibodies (1:1000). The exposure times for ab28670 were 10 s (rat) and 7 s (human), ab183103 were 30 min (rat) and 16 min (human), SC-100315 were 16 s (rat) and 6 s (human), and ab140703 was 6 min. Blots are representative of TG protein prepared from three different rats or two human cases.

Western blotting was performed to complement immunofluorescence and provide additional information about the potential PAC_1_ receptor presence within the TG. The SC-100315 anti-PAC_1_ receptor antibody was utilized for Western blotting as the non-specific band could be differentiated from other bands with MW. The ab28670, ab183103, and SC-100315 anti-PAC_1_ receptor antibodies detected several immunoreactive bands in protein from rat TG with varying intensities (Figure 4). Bands detected with different antibodies were of a similar but not identical MW (Figure 4, Table 2). Antibodies likely detect at least one form of the PAC_1_ receptor in TG at a size broadly consistent with the PAC_1_ receptor in transfected cell controls.

In protein from human TG, ab28670, ab183103, and the human-specific ab140703 all detected two similar immunoreactive smears (Figure 4, Table 2). The ab28670 antibody detected multiple additional bands, of which the higher MW band (~130–150 kDa) was also observed with ab140703. In contrast, many defined bands were detected with the SC-100315 anti-PAC_1_ receptor antibody, some of which were found at a similar MW to those observed with other antibodies (Figure 4, Table 2).

### 2.4. Characterization of Anti-VPAC_1_ and VPAC_2_ Receptor Antibodies

**Figure 5:**
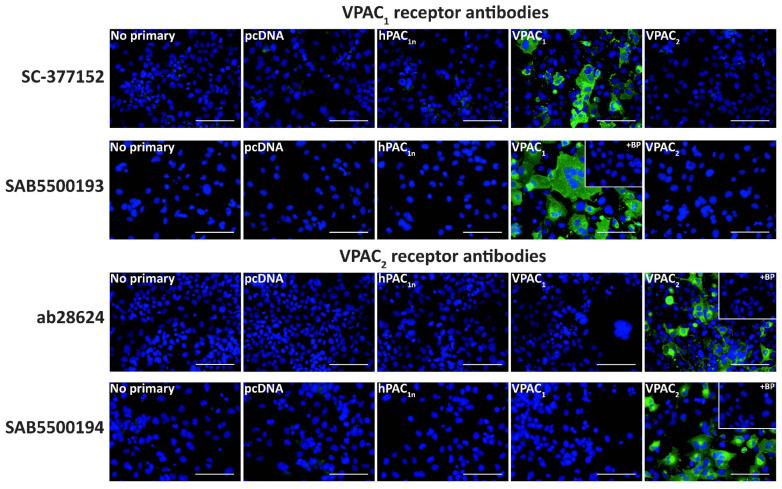
VPAC_1_ and VPAC_2_ receptor-like immunoreactivity detected in transfected Cos7 cells. Cells were transfected with the hPAC_1n_, hVPAC_1_ and hVPAC_2_ receptors or vector (pcDNA3.1^+^). Cells were stained with SC-377152 (1:200), SAB5500193, SAB5500194 (1:100, 1:200) or ab28624 (1:500, 1:1000). VPAC_1_ and VPAC_2_ receptor images shown were incubated with 1:100 SAB5500193 or SAB5500194, respectively and all other conditions with 1:200 antibody. The VPAC_2_ receptor image shown for ab28624 was incubated with 1:1000 antibody, and all other conditions with 1:500. Pre-absorbed blocking peptide (BP) antibody controls are presented as insets. Antibody immunoreactivity is shown in green and nuclear DAPI staining in blue. Images were taken with a 40× non-confocal objective and represent one field of view from three independent experiments. Scale bar, 100 µm.

A series of anti-VPAC_1_ and VPAC_2_ receptor antibodies were then characterized in transfected Cos7 cells to help elucidate whether VPAC receptor protein was present in the TG. The SC-377152 and SAB5500193 anti-VPAC_1_ receptor antibodies exhibited immunoreactivity in VPAC_1_ but not vector, PAC_1n_, or VPAC_2_ receptor transfected Cos7 cells (Figure 5, Table 1). Similarly, the ab28624 and SAB5500194 anti-VPAC_2_ receptor antibodies only detected immunoreactivity in cells transfected with the VPAC_2_ receptor (Figure 5, Table 1). Incubation of anti-VPAC_1_ or VPAC_2_ receptor antibodies with their BPs resulted in no detectable immunoreactivity. The SC-135604 anti-VPAC_2_ receptor antibody exhibited similar immunoreactivity in both vector and VPAC_2_ receptor transfected cells, even with antigen retrieval (Figure S6). This lack of robust staining suggests the antibody was unsuitable for use in immunofluorescence under the conditions tested in this study and was not used in further experiments.

**Figure 6:**
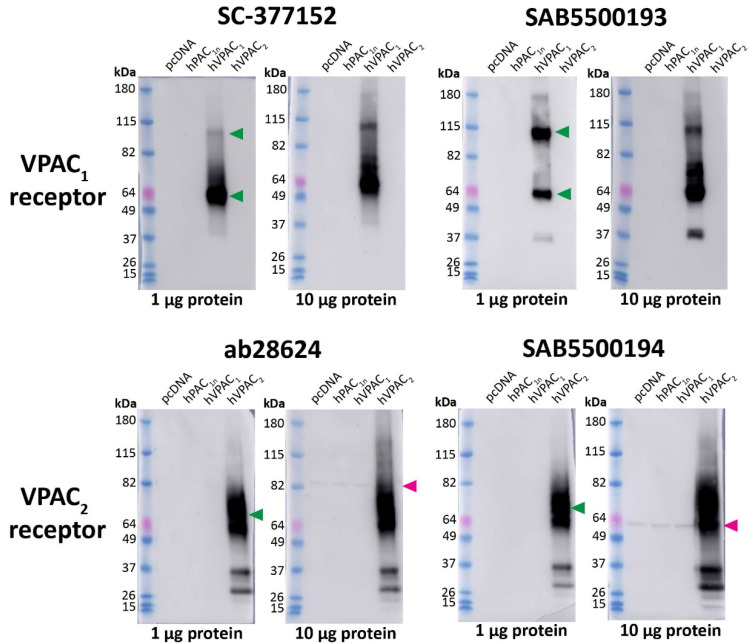
Apparent VPAC_1_ and VPAC_2_ receptor molecular weights detected by anti-VPAC receptor antibodies in transfected Cos7 cell protein. Protein samples were prepared from Cos7 cells transfected with the hPAC_1n_, hVPAC_1_ and hVPAC_2_ receptors or vector (pcDNA3.1^+^). Protein was loaded, and blots probed with anti-VPAC_1_ or VPAC_2_ receptor antibody (1:1000). The exposure times were 5 s (1 µg) and 3 s (10 µg) for SC-377152, 7 s (1 µg) and 3 s (10 µg) for SAB5500193, 2 s (1 µg) and 3 s (10 µg) for ab28624 and 15 s (1 µg) and 4 s (10 µg) for SAB5500194. Blots are representative of three (1 µg) or two (10 µg) independent experiments. Green arrowheads represent bands of an apparent MW consistent with those observed in a prior study using protein from transfected HEK293 cells [35]. A second green arrowhead indicates a receptor dimer. Pink arrowheads indicate major non-specific bands observed with 10 µg protein.

In Western blotting, the anti-VPAC_1_ receptor antibodies SC-377152 and SAB5500193 detected a large immunoreactive smear and a second higher MW band predicted to be a receptor dimer, consistent with the predicted sizes of the VPAC_1_ receptor (Figure 6, Table 1) [35]. However, additional bands were observed with SAB5500193. No immunoreactivity was observed in vector transfected, hPAC_1n_, or VPAC_2_ receptor protein samples with either antibody. The anti-VPAC_2_ receptor antibodies ab28624 and SAB5500194 detected a large immunoreactive smear which was consistent with the predicted size of the VPAC_2_ receptor, in addition to multiple lower MW bands (Figure 6, Table 1) [35]. Both anti-VPAC_2_ receptor antibodies detected a single band in the protein from vector, PAC_1n_, and VPAC_1_ transfected cells when 10 μg protein was loaded, as noted in Table S1 (Figure 6).

### 2.5. VPAC_1_ and VPAC_2_ Receptor-Like Immunoreactivity in Rat and Human TG

**Figure 7:**
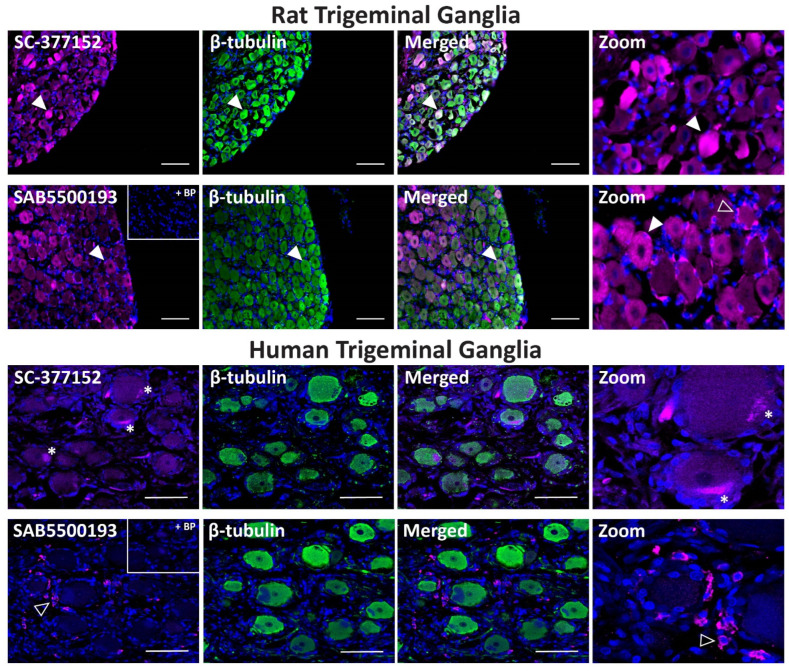
VPAC_1_ receptor-like immunoreactivity detected in rat and human trigeminal ganglia. VPAC_1_ (SC-377152, SAB5500193, 1:100) receptor-like immunoreactivity in TG neurons and glia colocalized with β-tubulin (1:500). Pre-absorbed blocking peptide (BP) antibody controls are presented as insets. Receptor immunoreactivity is shown in pink, β-tubulin in green, colocalized regions appear white, and DAPI (rat) or Hoechst (human) stained nuclei in blue. Solid arrowheads indicate examples of immunoreactive neurons and empty arrowheads examples of immunoreactive glia. Images were taken with a 20× non-confocal objective and are representative of TG staining in three individual rat or human cases. Scale bar, 100 µm. * autofluorescence due to lipofuscin.

In rat TG, anti-VPAC_1_ receptor antibodies exhibited strong immunoreactivity in some neuronal cell bodies and glial-like cells, especially with SAB5500193 (Figure 7, Table 2). Occasional colocalization with β-tubulin was observed in neurons for both antibodies. Preincubation of SAB5500193 with its BP abolished both neuronal and glia-like staining. In human TG, no immunoreactivity was observed with the SC-377152 VPAC_1_ receptor antibody (Figure 7). In contrast, the SAB5500193 VPAC_1_ receptor antibody stained occasional glial-like cells, which was abolished in the presence of BP (Figure 7, Table 2).

**Figure 8:**
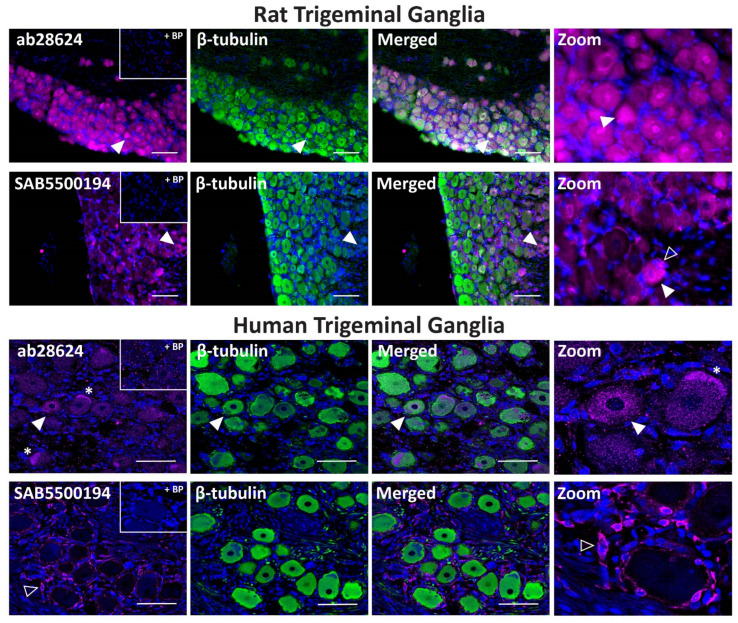
VPAC_2_ receptor-like immunoreactivity detected in rat and human trigeminal ganglia. VPAC_2_ (ab28624, 1:500, SAB5500194, 1:100) receptor-like immunoreactivity in TG neurons and glia colocalized with β-tubulin (1:500). Pre-absorbed blocking peptide (BP) antibody controls are presented as insets. Receptor immunoreactivity is shown in pink, β-tubulin in green, colocalized regions appear white, and DAPI (rat) or Hoechst (human) stained nuclei in blue. Solid arrowheads indicate examples of immunoreactive neurons and empty arrowheads examples of immunoreactive glia. Images were taken with a 20× non-confocal objective and are representative of TG staining in three individual rat or human cases. Scale bar, 100 µm. * autofluorescence due to lipofuscin.

The ab28624 antibody exhibited weak VPAC_2_ receptor-like immunoreactivity relative to background in all rat TG neuronal cell bodies, with occasional neurons showing more intense staining (Figure 8, Table 2). The neuronal staining of SAB5500194 was more pronounced in independent experiments, but consistent glia-like staining was detected (Figure 8, Table 2, Figure S3). Preincubation of ab28624 and SAB5500194 with their BPs abolished both neuronal and glia staining. In human TG, VPAC_2_ receptor-like immunoreactivity was observed in some neuronal cell bodies with ab28624 (Figure 8, Table 2). However, this antibody exhibited high background staining. Furthermore, preincubation with its BP resulted in variable changes in background immunoreactivity and lipofuscin intensity (Figure 8, Figure S4). The SAB5500194 antibody exhibited VPAC_2_ receptor-like immunoreactivity in glial-like cells surrounding most neurons, which was abolished in the presence of BP (Figure 8, Table 2).

### 2.6. Evaluation of VPAC_1_ and VPAC_2_ Receptor Antibodies with Western Blotting in Rat and Human TG

**Figure 9:**
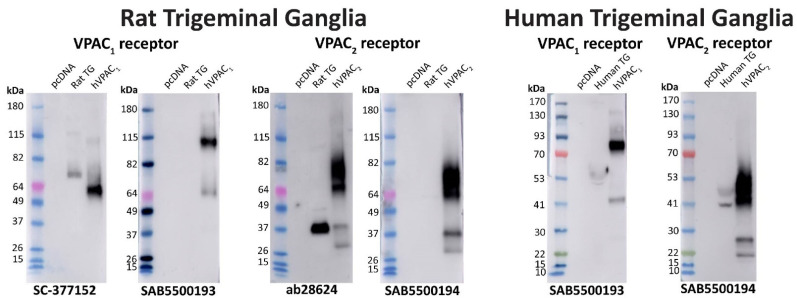
VPAC_1_ and VPAC_2_ receptor apparent molecular weights detected in rat and human trigeminal ganglia. Protein samples were prepared from TG or Cos7 cells transfected with the human PAC_1n_, VPAC_1_ and VPAC_2_ receptors or vector (pcDNA3.1^+^_)_. Protein was loaded, and blots probed with anti-VPAC receptor antibodies (1:1000). The exposure times for SC-377152 was 16 s, SAB5500193 were 8 min (rat) and 3 min (human), ab28624 was 14 s, and SAB5500194 were 15 s (rat) and 8 s (human). Blots are representative of TG protein prepared from three different rats or two human cases.

Two immunoreactive bands were detected with the SC-377152 anti-VPAC_1_ receptor antibody in protein from rat TG (Figure 9, Table 2). In contrast, the SAB5500193 antibody failed to detect any clear immunoreactivity, even after longer exposure (Figure 9). The ab28624 anti-VPAC_2_ receptor antibody detected a strongly immunoreactive band and several weaker bands in protein from rat TG (Figure 9, Table 2). In contrast, the SAB5500194 antibody detected a single non-specific band but no additional bands, even following longer exposure (Figure 9, Table 2). In protein from human TG, a single weakly immunoreactive band was observed with the anti-VPAC_1_ receptor antibody (SAB5500193) and two immunoreactive bands were detected by the anti-VPAC_2_ receptor antibody (SAB5500194) (Figure 9, Table 2).

## 3. Discussion

Effectively targeting the PACAP system for treating migraine and headache disorders requires identifying where the PAC_1_, VPAC_1_, and VPAC_2_ receptors are expressed. Validated, well-characterized anti-receptor antibody tools are a necessity to understand receptor expression. The current suite of anti-PACAP-responsive receptor antibodies are poorly characterized, difficult to obtain, or are no longer available. To bridge this gap, we characterized several commercial antibodies targeting the PAC_1_, VPAC_1_, and VPAC_2_ receptors. Multiple strategies were then used to examine their specificity and ability to detect physiological protein levels in the TG.

An Important antibody validation tool to help detect off-target antibody immunoreactivity is tissue from gene knockout animals [26]. PAC_1_, VPAC_1_, and VPAC_2_ receptor null mouse models have been generated [44,45,46,47,48]. However, many null mice models are not global knockouts but rather exon deletions corresponding to receptor transmembrane regions. This means that the N- and C-terminal regions targeted by the anti-PACAP-responsive receptor antibodies in the current study may still produce reactivity in these knockout models, even when no functional receptor is present. Therefore, transfected cells as a genetic model, especially the vector negative control, are essential for validating antibodies against the PACAP receptor family.

The anti-PAC_1_ receptor antibodies used in this study, including the commonly used ab28670, had not been previously validated using a genetic strategy. We identified several anti-PAC_1_ receptor antibodies as suitable for immunofluorescence in overexpressing receptor cell models as they detected the protein of interest robustly and exhibited no immunoreactivity in negative controls. This was similar for most anti-VPAC_1_ and VPAC_2_ receptor antibodies, consistent with prior immunofluorescence studies in transfected HEK293 cells using the recombinant anti-VPAC_1_ (SAB5500193) and VPAC_2_ (SAB5500194) receptor antibodies [35,49]. The current study also used BPs, where the antigen was known, to show that PACAP-responsive receptor antibodies appear to recognize and bind their target antigen. This control is crucial for polyclonal antibodies supplied as whole antisera, such as ab28670 (PAC_1_) and ab28624 (VPAC_2_), which may contain other antibodies that recognize off-target antigens. However, an antibody that can detect the antigen it was raised against may still recognize and bind with high affinity to other non-specific epitopes that have a similar shape or sequence to the intended epitope [36,50]. Therefore, this needs to be considered when using these antibodies in biological samples, and additional methods, such as Western blotting, are useful to examine additional non-specificity based on MW.

Western blotting is a well-used and beneficial additional antibody validation method [23,24]. Overall, the PAC_1_ antibodies detected major immunoreactive bands in human and rat receptor membranes with apparent MWs consistent to those observed in previous studies using antibodies or tagged receptors [49,51,52]. The similarity in MW between the different antibodies detected with Western blotting, in addition to the small shift in size detected between the PAC_1n_ and PAC_1s_ receptors, indicates that the antibodies tested recognize proteins likely to be the PAC_1_ receptor. The anti-VPAC_1_ and anti-VPAC_2_ receptor antibodies also detected major immunoreactive bands that were of a consistent size to those previously reported in the literature [35,49]. However, many of the PACAP-responsive receptor antibodies identified additional bands that were not in the vector control nor seen in prior studies using transfected cells. These bands could be due to the use of different cell lines or methods that may have additional exposed epitopes the antibody may bind to, or could relate to intermediates in the receptor synthesis or degradation pathways, as these were membrane-enriched samples [23,36]. Most antibodies also exhibited non-specific bands not reported in prior studies, primarily when higher protein amounts were loaded. This emphasizes the usefulness of a vector control as a substitute for KO tissue in addition to characterizing antibodies with a range of protein amounts to determine any potential non-specificity, which can be significant sources of off-target staining in tissues [29]. However, as most of these non-specific bands were clearly distinguishable from the target protein band, the antibodies characterized have some utilization for both fluorescent ICC and western blotting in transfected cell systems with the inclusion of appropriate controls.

The current study identified the first anti-PAC_1_ receptor antibody (ab140703) that can distinguish between splice variants, specifically between the human PAC_1n_ and N-terminally truncated PAC_1s_ receptor. The differential expression of receptor splice variants is one possible cause of the variability in efficacy and adverse effects exhibited by drugs targeting GPCRs [32,53,54,55]. Therefore, it is important to understand the localization of receptor variants in tissues of interest. However, this is limited by the lack of splice variant-specific antibodies available, largely due to homology between variants leading to selectivity issues [54]. Additionally, very few studies have considered different receptor splice variants during antibody validation. Examining human PAC_1n_ (full N-terminus) expression using the variant-specific antibody identified in this study may help clarify potential differences between these receptor variants and lead to a better understanding of their role in migraine and headache disorders. However, this antibody is not able to determine whether the receptor detected contains a Hop, Hip, or no inserted cassette within the ICL3. Furthermore, this antibody appears to be most useful for the detection of PAC_1_ receptors in histological studies where endogenous expression is relatively high over background or in Western blotting.

Protein expression of PACAP-responsive receptors in the TG has been examined, but the current study is the first to investigate these findings using commercial antibodies validated through a genetic approach. Western blotting suggested that a PAC_1_ receptor variant was present in the TG as multiple antibodies detected proteins of a similar MW. Furthermore, detection of a band with the ab140703 antibody proposes that the PAC_1n_ variant is among those expressed in the TG, consistent with previous mRNA and pharmacological studies [21,56]. When looking at the cellular localization of PAC_1_, the current study found PAC_1_ receptor expression in TG neurons similar to several previous studies [33,39,40]. However, contrasting with prior literature, one study using a transfected cell validated the antibody reported no PAC_1_ immunoreactivity and only limited mRNA expression in the TG [38]. This antibody, although specific, may not exhibit sufficient sensitivity or requires specific experimental conditions to detect endogenous levels of PAC_1_ receptor protein. A second study has also reported a lack of neuronal PAC_1_ staining, and instead saw PAC_1_ receptor-like immunoreactivity in TG glia [41]. However, this contradicts an earlier study published by the same group using the same ab28670 antibody [33,41]. This discrepancy emphasizes how variable antibodies can be, especially those supplied as whole serum polyclonal antibodies, like ab28670, which are subject to potentially significant lot-to-lot variation. Furthermore, the difficulty in detecting neuronal PAC_1_ expression, as observed between these studies and the human TG within the current study, indicates that PAC_1_ may be expressed at relatively low levels or not consistently expressed within these structures and may differ vastly between cases. Interestingly, within the current study, PAC_1_ receptor-like immunoreactivity was also found occasionally in TG glia, consistent with a recent report [41]. This expression is further supported by prior reports of PACAP activity in isolated rat TG glia and suggests the PAC_1_ receptor may play a role in glia- and neuronal-driven functions [56,57].

Despite clear PAC_1_ receptor-like immunoreactivity in the TG within the current study, the tested antibodies displayed limitations in tissue, which made interpretation difficult. Differences in cellular localization between antibodies and species were observed, likely related to the individual antibody avidity or sample processing methods; both factors are known to affect high-affinity epitope availability [24,58]. Western blotting using TG was also difficult to interpret, especially for the anti-PAC_1_ antibodies, and we cannot explain why different antibodies targeting regions present in all known receptor variants do not always detect the same pattern of bands. For this reason, we cannot definitely conclude which bands are PAC_1_ even with the use of transfected cell controls as a guide. This difference could relate to tissue-dependent receptor splice expression or antibodies recognizing an epitope that could be masked or exposed in a post-translationally modified form and, therefore, detects different-sized proteins; however, this has not been specifically examined, and future studies could include mass spectrometry to further investigate this [58,59,60]. Conclusively determining if these antibodies yield genuine receptor-driven immunoreactivity is difficult without the use of knockout models. Therefore, further characterization of these antibodies may be required in endogenous tissues. Despite these limitations, the tested anti-PAC_1_ receptor antibodies may have use in a species- and tissue-dependent context where higher protein levels are present.

Pharmacological and mRNA-based approaches suggest that PAC_1_, rather than VPAC_1_ and VPAC_2_, is predominantly expressed in the TG [2,20,21,56,61]. In contrast, and in line with the limited histological and Western blotting data available, the current study observed pronounced immunoreactivity of the VPAC_1_ and VPAC_2_ receptor in the TG [33,40,43]. Immunoreactivity was predominantly found in TG glia consistent with a previous study, but neuronal staining in rats was also observed in the current study, suggesting they may play a wider role within the TG than previously predicted [33]. However, these antibodies exhibited similar limitations to PAC_1_, whereby differences between species and distinct antibodies were observed likely due to differences in avidity. Overall, the anti-VPAC_1_ and VPAC_2_ antibodies, especially the recombinant monoclonals, are promising tools for future research.

The success of monoclonal anti-CGRP receptor antibody therapeutics as migraine treatments has led to an interest in developing a similar therapeutic targeting the PACAP system. However, the lack of clinical efficacy with AMG301, an anti-PAC_1_ receptor antibody, suggests that the PAC_1_ receptor, or the specific variant it targets may not be important or expressed in relevant cell types [18,19]. It would be interesting to examine PAC_1_ expression using AMG301 in our models to determine whether it detects specific PAC_1_ splice variants. Alternatively, there may be redundancy in the mechanisms underlying migraine pathogenesis and targeting PAC_1_ alone may not be sufficient to treat this disease in most patients. Interestingly, the PAC_1_ receptor was initially identified as a potential target for migraine and headache disorders as infusion of PACAP, but not VIP, produced migraine-like symptoms [7,8,62]. More recently, sustained infusion of VIP reportedly produced delayed headache and migraine-like symptoms [10,13]. The expression pattern observed for both VPAC_1_ and VPAC_2_ receptors in the TG suggests that the delayed VIP activity may involve satellite glial cells, which have been implicated in peripheral and central sensitization [63]. Furthermore, the TG also expresses mRNA encoding the PAC_1s_ receptor splice variant, which can be potently activated by VIP and may be involved in this phenomenon [15,21,64]. In addition to investigating the role of different receptor subtypes and splice variants in migraine, future studies utilizing these characterized antibodies could include whether PACAP or VIP knockout stimulates the differential expression of receptor subtypes in migraine-related structures. Furthermore, examining the relationship between these receptors and others regulated by PACAP and involved in migraine, such as the CGRP and serotonin receptors, would also be of interest [65,66].

Research examining the role of migraine-relevant proteins in human tissue has primarily been conducted in healthy tissues [4,5,6,20,33,38]. This is likely due to difficulties in obtaining a consistent group of diseased tissues from patients. Migraine is a diverse disorder that is characterized by a range of triggers, symptoms, and thus potential mechanisms. Furthermore, specific migraine subtypes, such as the menstrual migraine or familial hemiplegic migraine, may have differences in symptoms, frequency, and resolution with age [67]. Similarly, headache disorders include a huge range of conditions, ranging from tension headache to trigeminal neuralgia [68]. The potential for differential regulation of PACAP-responsive receptors between healthy and diseased tissues should also be considered. The antibodies within this study could be used to compare PACAP-responsive receptor expression between the tissue from healthy individuals and patients suffering from migraine subtypes or specific headache disorders. This would allow tissue- or cell-dependent receptor regulation to be examined and provide further insight into their role in these disorders.

This study characterized multiple commercially available antibodies targeting the PAC_1_, VPAC_1_, and VPAC_2_ receptors. All the tested antibodies, except one, detected their target protein in transfected cells. However, when used to examine endogenously expressed PACAP-responsive receptors in the TG, antibodies produced different patterns of immunoreactivity. This is likely due to differences in sensitivity, which made interpretation difficult. Despite this, antibodies, especially the anti-VPAC_1_ and VPAC_2_ antibodies, could be used to inform the expression pattern of PACAP-responsive receptors in tissues with careful scrutiny, the use of multiple strategies, and the incorporation of several controls. The presence of VPAC_1_ and VPAC_2_, in addition to PAC_1_ receptors within TG neurons and glia, suggests all three receptor subtypes play important roles in multiple cell types and represent promising targets for developing migraine and headache disorder treatments.

## 4. Materials and Methods

### 4.1. Antibodies and Blocking Peptides

Multiple antibodies, targeting different regions of human PAC_1_, VPAC_1_, and VPAC_2_ receptors were selected. These included commonly used tools, those that looked promising based on limited reports, manufacturers descriptions, or the potential to differentiate between splice variants (Table S1). The haemagglutinin (HA) antibody was purchased from Biolegend (#901503, San Diego, CA, USA, RRID: AB_256005) and anti-β-tubulin from Sigma Aldrich (#T2200, Missouri, MO, USA, RRID: AB_262133). Where the antigenic sequence was known, blocking peptides (BP) corresponding to this sequence were synthesized in-house (Supporting Information, Table SC1, Schemes SC1–4, Figures SC1–8).

### 4.2. HA-Tagging of Human PAC_1_ Receptors

A complementary set of primers were designed using NEBaseChanger to insert a single HA-tag (YPYDVPDYA) at Cys^25^ of the human PAC_1n_ and PAC_1s_ receptors. This position was chosen as it was predicted to not affect ligand binding and is immediately following the signal peptide. The primer sequences were as follows: forward primer, GCCGGATTATGCGATCTTCAAGAAGGAGCAAGC, and reverse primer, ACATCATACGGATAGCAGTCAGAATGCATGGC. Site-directed mutagenesis was performed using the Q5^®^ site-directed mutagenesis kit. Briefly, 20 ng of the PAC_1_ receptor DNA was added to 10 μL Q5 master mix, 0.5 μM each of the forward and reverse primers, and nuclease-free water to a final volume of 20 μL. Using a PCR thermocycler, the DNA was denatured at 95 °C for 2 min, followed by 25 cycles of denaturation at 98 °C for 10 s, annealing at 64 °C for 15 s, and extension at 72 °C for 3.5 min. To allow circularisation, 1 μL of the PCR product was added to 1 μL Kinase-Ligase-DpnI (KLD) enzyme mix, 5 μL KLD reaction buffer and diluted in nuclease-free water to a final volume of 10 μL. This solution was incubated at room temperature for 5 min. Following incubation, the DNA was transformed into XL.10-gold Ultracompetent *E. coli*, purified using the NucleoBond Xtra Maxi kit, and sequenced prior to use. The HA-tagged PAC_1_ receptors are known to be functional and exhibit similar behaviour to their un-tagged counterparts for cAMP accumulation (Figure S8).

### 4.3. Cos7 Cell Culture and Transfection

Culture and transient transfection of Cos7 cells were performed as previously described [15,69]. The cells were seeded at 20,000 cells/well into 96-well CellCarrier ultra plates (PerkinElmer; Waltham, MA, USA) for ICC experiments or ~5 million cells in 150 mm culture dishes for western blotting. The human PAC_1n_, PAC_1s_, VPAC_1_, and VPAC_2_ receptor constructs in pcDNA3.1 were as previously described [15]. The rat PAC_1n_ receptor was purchased from GenScript (Piscataway, NJ, USA). The rat PAC_1s_ was generated in-house.

### 4.4. Immunofluorescence in Transfected Cos7 Cells

The transfected Cos7 cells were fixed with 4% paraformaldehyde then washed with PBS. For N-terminally directed antibodies, antigen retrieval was performed in a 10 mM Tris-sodium citrate buffer containing 0.05% Tween (pH 6.0). The cells were blocked in 10% goat serum for 1 h at RT. The well contents were replaced with primary antibody (Table S1) for 30 min at 37 °C or 4 °C overnight (N-terminal antibodies). Under some conditions, a primary antibody was pre-incubated with an excess (five times antibody mass (g)) of BP for 90 min at 20 °C. The cells were washed and incubated with secondary antibody (Table S2) and DAPI for 1 h at RT in the dark and imaged using an Operetta high content imager (PerkinElmer) with a 40× non-confocal 0.6NA LWD objective lens on enhanced mode (gamma value of 1.52). Each condition was performed in duplicate wells and at least 5 fields of view were imaged per well. A secondary antibody only (no primary) and vector (pcDNA3.1^+^) control were included in each independent experiment.

### 4.5. Rat and Human Trigeminal Ganglia Tissue

TG was obtained from adult Sprague-Dawley (SD) rats as detailed in Table S3 as part of routine colony maintenance. The animals were housed in Tecniplast Conventional 1500U cages in an enriched environment under a 12/12 h light/dark cycle at 22 ± 2 °C. The rats had *ad libitum* access to water and standard chow (Teklad TB 2018, Madison, WI, USA). All animal care and procedures were conducted in accordance with the New Zealand Animal Welfare Act (1999) and approved by the University of Auckland Animal Ethics Committee and are reported in accordance with the ARRIVE 2.0 guidelines [70,71]

Post-mortem human TG (Table S3) were obtained as part of the University of Auckland Human Body Bequest Program for teaching and research. All procedures involving the use of human tissue were conducted in accordance with the Human Tissue Act (2008). Fresh-frozen human TG was obtained from the National Institute of Health NeuroBio Bank (USA) (Table S3).

### 4.6. Western Blotting

The transfected Cos7 cells in 150 mm dishes were harvested in PBS using a cell scraper and pelleted at 4000× *g* for 10 min at 4 °C. For the preparation of membrane-enriched protein samples, pelleted Cos7 cells or rat TG tissue were sonicated (Qsonica Q700, amplitude 10, pulse 1 s on/off for 1 min) in Tris-NaCl solubilization buffer (pH 8.0) containing a complete mini EDTA-free protease inhibitor cocktail (1:10,000; Roche Applied Science, IN, USA). Sonicated samples were centrifuged (30,000× *g*, 30 min, 4 °C) and resuspended in solubilization buffer containing 1% n-dodecyl-β-D-maltopyranoside (DDM)/0.1% cholestryl hemisuccinate tris salt (CHS) and left to solubilize for 2 h at 4 °C on an end-over-end homogenizer. To collect the soluble membrane fraction, samples were centrifuged (30,000× *g*, 30 min, 4 °C) and the supernatant aliquoted into protein Lo-bind tubes (Eppendorf, Hamburg, Germany) and stored at −80 °C.

To prepare human TG whole tissue lysates, tissues were homogenized in RIPA buffer (Tris-NaCl pH 8.0, 0.1% SDS, 0.5% sodium deoxycholate, 1% Triton X-100) containing a protease inhibitor cocktail (1:10,000; Roche Applied Science) using a 1 mL glass homogenizer. The samples were left to solubilize for 2 h at 4 °C on an end-over-end homogenizer. The samples were pelleted by centrifugation (16,000× *g*, 20 min, 4 °C), and the supernatant was aliquoted into protein Lo-bind tubes and stored at −80 °C. The protein concentration for all samples were quantified using a Pierce BCA protein assay kit (ThermoFisher Scientific, Waltham, MA, USA).

The protein samples were diluted in solubilization or RIPA buffer and denatured with a 4× loading dye (2.5 mL 1M Tris-HCL, 4 mL 20% sodium dodecyl sulfate, 4 mL 100% glycerol, 0.04 mg bromophenol blue) and 0.1M DTT for 1 h at 37 °C. When required, the samples were incubated in the presence of 1 µL PNGaseF (New England Biolabs, Ipswitch, MA, USA) for an additional hour at 37 °C prior to incubation with the loading dye. The protein (0.1–50 μg, Table S4) was loaded onto 4–12% SurePage Bis-Tris gels (GenScript, NJ, USA) alongside a protein ladder (Benchmark; Life Technologies, Carlsbad, CA, USA, Cat# 10,748,010 or Abcam, Cambridge, UK, Cat # ab116027) and run in MOPS buffer at 180 V. The proteins were then transferred to a 0.45 µm nitrocellulose membrane at 100 V for 45 min. The blots were blocked with 5% non-fat milk in TBS containing 0.1% Tween20 (TBS-T) for 1 h at room temperature before addition of primary antibody diluted to 1:1000 in 5% milk/TBS-T for 1 h at room temperature (anti-HA, 1:2000) or overnight at 4 °C (receptor antibodies, Table S1). The blots were then washed twice and incubated with secondary antibody (Table S2, 1:10,000) in 5% milk/TBS-T for 1 h at room temperature. The blots were washed a further two times and developed using Supersignal west pico Plus ECL reagent (ThermoFisher Scientific), and the chemiluminescent signal was visualized using an Amersham Imager A600 initially using the automated exposure function and then adjusted manually (GE Healthcare, Chicago, IL, USA).

### 4.7. Immunofluorescence in Rat and Human Trigeminal Ganglia

The processing and immunofluorescent staining of rat and human trigeminal ganglia was performed as described previously with minor modifications [72]. Briefly, rats were euthanized by asphyxia under CO_2_ inhalation, followed by cervical dislocation. The TG were dissected and washed in PBS before overnight fixation in 4% PFA at 4 °C. The tissues were washed in PBS, cryoprotected in 20% sucrose (*w*/*v*) overnight, and embedded in optimal cutting temperature compound (Sakura Tissue-Tek, Torrance, CA, USA, #4583) using liquid nitrogen. The TG tissue was then sectioned sagitally at 12 µm using a Leica CM1850 microtome (Leica Biosystems, Wetzlar, Germany). The sections were mounted onto slides and stored at −20 °C. The human TG were dissected from formalin-fixed cadavers and dehydrated in a series of 60–100% ethanol solutions then cleared in xylene and paraffin embedded. The human TG were sectioned sagitally at 10 µm on a rotary microtome (Leica Biosystems, HI 2235), floated in a 38 °C water bath, and mounted onto slides. The sections were dried overnight and stored at RT until use.

Following the deparaffinization and hydration of the human TG sections, both rat and human TG were immersed in a 10 mM Tris-sodium citrate buffer containing 0.05% Tween20 (pH 6.0) and placed in a microwave (rat TG) or pressure cooker (human TG) for antigen retrieval. The tissues were then washed in TBS-T (0.1% Tween20 for the rat sections, 0.05% Tween20 for the human sections), blocked in 10% goat serum/TBS-T for 1 h at room temperature, and incubated with primary antibody/1% serum in TBS-T (Table 1) in the presence or absence of BP and the neuronal marker β-tubulin (1:500) overnight at 4 °C. The sections were washed and incubated with secondary antibody/1% serum in TBS-T (Table S1, 1:500) for 1 h (rat TG) or 3 h (human TG) at room temperature. Sections were washed a final time and cover-slipped using the ProLong Gold mounting medium (Life Technologies). For both the rat and human TG, serial sections were not used for staining.

The sections were imaged using an Operetta 20× non-confocal 0.75 high NA objective lens (gamma value of 1) (rat) or a Zeiss LSM 710 inverted confocal microscope (Zeiss, Oberkochen, Germany) with a 20× 0.8NA objective lens (human). TG sections demonstrated no background staining due to autofluorescence or secondary antibody in the absence of a primary antibody (Figure S9).

### 4.8. Image Analysis and Adjustment

PNG files (ICC) or raw TIFF files (16-bit, rat TG) were acquired from the Harmony 4.1 software on the Operetta. Raw TIFF files (human TG) were obtained from the Zeiss ZEN 2010 software as 12-bit LSM files. The images were then pseudocolored; were adjusted for color, brightness, and contrast; were merged; and had scale bars added in Image J (v1.53k). Care was taken to avoid misrepresentation due to the loss of data by over-adjusting the brightness and contrast as per the recommendations for reproduceable and accurate reporting of microscopy data [73,74]. Any adjustments were applied uniformly across the whole image and across all conditions for an antibody.

For all figures, representative images are presented from three independent experiments using separate antibody dilutions and tissue from different rats or human cases. The exception to this was the human TG Western blotting, which had two. Figures presented in the main manuscript are from the rat #18, human case #15A (IHC), and rat #41, human TG #1322 (Western blotting).

## Figures and Tables

**Table 1:** Summary of PACAP-responsive receptor antibody characterization in transfected Cos7 cells.

Antibody		ICC	Western Blotting			
		Specificity *	Specificity	Detected Bands (~kDa)	Bands Consistent with Target ^a^ (~kDa)	Bands in pcDNA (~kDa)
anti-PAC_1_	ab28670	+++ *	++	28, 40–65, 95–130	40–65, 95–130	28, 33, 53
	ab183103	+++ *^$^	++	40–60, 93–130	40–60, 95–130	55, 68
	ab140703	+++ ^^$^	+++ ^	50, 53–60, 93–130	50, 53–60, 95–130	93
	SC-100315	+++ ^$^	++	40–68, 95–130	40–65, 95–130	68
anti-VPAC_1_	SC-377152	+++	+++	40–55, 70	55, 70	None
	SAB5500193	+++	++	28, 40–55, 70–75, 130	55, 70–75	None
anti-VPAC_2_	ab28624	+++	++	22, 28, 40–55	50–55	60
	SAB5500194	+++	++	22, 28, 40–65	50–65	41
	SC-135604	-	ND	ND	ND	ND

The antibody specificity indicates the antibody’s ability to detect the target protein without detecting other related receptors and is described as -, +, ++ or +++. No visible immunoreactivity for the target protein, similar to that observed with pcDNA is indicated with a dash (-). + indicates strong immunoreactivity unrelated to the target protein. Immunoreactivity that appears to be specific but exhibits weak immunoreactivity for unrelated proteins is indicated with ++ and +++ indicates immunoreactivity that appears to be specific to the target protein and not unrelated proteins. * For antibodies raised against a known antigenic sequence, immunoreactivity was reduced in the presence of blocking peptide. ^ detects only the human PAC_1_ receptor with a full N-terminal domain. ^$^ required antigen retrieval. To provide accurate receptor sizes, the apparent molecular weights were based on the interpretation of the approximate size of the protein using the comparison between Benchmark and Abcam protein ladders. ^a^ molecular weight consistent with the apparent size of the human target receptor from the control HA-PAC_1_ receptor blot (Figure S1) or VPAC receptor blots from Schulz et al., 2015. ND = not determine.

**Table 2:** Summary of PACAP-responsive receptor-like immunoreactivity in rat and human TG.

Antibody		Rat Trigeminal Ganglia			Human Trigeminal Ganglia		
		Immunofluorescence		Western Blotting	Immunofluorescence		Western Blotting
		Level of Immunoreactivity	Neurons/Glia	Bands Detected (~kDa)	Level of Immunoreactivity	Neurons/Glia	Bands Detected (~kDa)
anti-PAC_1_	ab28670	+++ *	Neurons	25, 32, 34, 45, 60, 65, 80, 85, 120, 130	+^#^ *	Glia	26, 35, 41, 48, 53–65, 95, 130, 170
	ab183103	+ *	Neurons	25, 32, 34, 45, 50, 60, 65, 80	+^#^ *	Neurons	53–65, 85
	ab140703 ^	ND	ND	ND	++/+	Neurons, glia (1 case)	25–28, 50–65, 85–100, 120–130
	SC-100315	ND	ND	50–60, 68	ND	ND	48, 52, 68, 120, 130–165
anti-VPAC_1_	SC-377152	+++	Neurons, glia	50, 85	-	-	ND
	SAB5500193	+++ *	Neurons, glia	-	+^#^ *	Glia	53–65
anti-VPAC_2_	ab28624	++ *	Neurons	28, 35, 48, 70	+^#^	Neurons (1 case)	ND
	SAB5500194	+^#^/+++ *	Neurons, glia	-	+++ *	Glia	41, 45–50

The antibody level of immunoreactivity and prevalence is specified. No visible immunoreactivity is indicated with a dash (-). + indicates weak immunoreactivity in very few cells. +^#^ indicates intense immunoreactivity in very few cells. Intense immunoreactivity that was occasionally observed in cells is indicated with ++, +++ indicates high intensity immunoreactivity that was observed in many cells. * For antibodies raised against a known antigenic sequence, immunoreactivity was reduced in the presence of blocking peptide. ^ detects only the human PAC_1_ receptor with a full N-terminal domain. ND = not determined. To provide accurate receptor sizes, the apparent molecular weights in rat TG blots were based on the interpretation of the approximate size of the protein using the comparison between Benchmark and Abcam protein ladders.

## Data Availability

The data that support the findings of this study are available from the corresponding author upon reasonable request.

## Supplementary Materials

The following supporting information can be downloaded at: https://www.mdpi.com/article/10.3390/ijms232213797/s1.
