# Supplementary material for: PAC1, VPAC1, and VPAC2 Receptor Expression in Rat and Human Trigeminal Ganglia: Characterization of PACAP-Responsive Receptor Antibodies
Source: Int J Mol Sci. 2022 Nov 9;23(22):13797. doi: 10.3390/ijms232213797 (PMC9697343; doi:10.3390/ijms232213797)
Supplement: Supplementary file 1 [file ijms-23-13797-s001.zip › ijms-1970803-supplementary.pdf]

Supporting Information

PAC_1, $VPAC_1$ and $VPAC_2$ receptor expression in rat and human trigeminal ganglia: characterization of PACAP-responsive receptor antibodies

Zoe Tasma[1], Andrew Siow[1,2], Paul W. R. Harris[1,2,3], Margaret A. Brimble[1,2,3], Simon J. O'Carroll[4], Debbie L. Hay[3,5], Christopher S. Walker[1,3*]

[1]School of Biological Sciences, The University of Auckland, Auckland, New Zealand.

[2]School of Chemical Sciences, The University of Auckland, Auckland, New Zealand.

[3]Maurice Wilkins Centre for Molecular Biodiscovery, The University of Auckland, Auckland, New Zealand.

[4]Department of Anatomy and Medical Imaging and Centre for Brain Research, Faculty of Medical and Health Science, The University of Auckland, Auckland, New Zealand

[5]Department of Pharmacology and Toxicology, The University of Otago, Dunedin, New Zealand

*Corresponding author: Christopher S Walker, E-mail: cs.walker@auckland.ac.nz

Supporting Tables

Table S1. Characteristics of PACAP-responsive receptor antibodies.

Antibody (clone)	Host/clonality	Immunogen		Purification	Reactivity	Supplier
**PAC_1 receptor**						
**ab28670**	Rabbit polyclonal IgG	aa 448-468 of $hPAC_1$ LSKSSSQIRMSGLPADNLAT	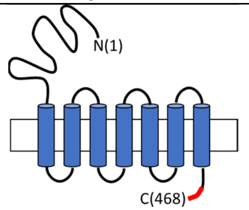	Whole serum	H (R, M)	Abcam, Cambridge, UK RRID: AB_777009
**ab183103**	Rabbit polyclonal IgG	aa 34-47 of $hPAC_1$ CLEKIQRANELMGF	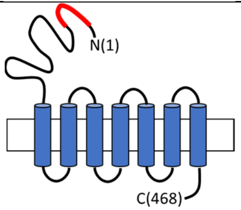	Affinity purified	H, M	Abcam, Cambridge, UK No RRID
**ab140703**	Rabbit polyclonal IgG	N-terminus of $hPAC_1$	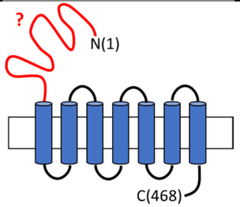	Affinity purified	H	Abcam, Cambridge, UK No RRID
**SC-100315 (1B5)**	Mouse monoclonal IgG_1	Unknown	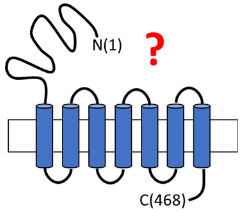	Unknown	H	Santa Cruz Biotechnology, CA, USA RRID: AB_1126992
**$VPAC_1$ receptor**						

**SC-377152 (B4)**	Mouse monoclonal IgG_{2a}	aa 31-160 of $hVPAC_1$	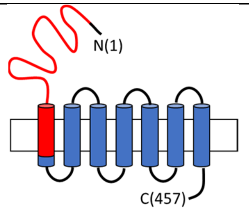	Unknown	H	Santa Cruz Biotechnology, CA, USA No RRID
**SAB5500193 (SP234)**	Rabbit monoclonal IgG	aa 438-457 of $hVPAC_1$ TRVSPGARRSSSFQAEVSLV	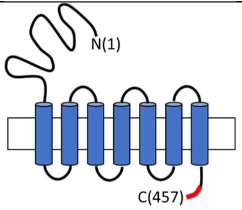	Protein A/G	H, R (M)	Sigma-Aldrich, MO, USA No RRID
**$VPAC_2$ receptor**						
**ab28624**	Rabbit polyclonal IgG	aa 419-438 of $hVPAC_2$ LQFHRGSRAQSFLQTETSVI	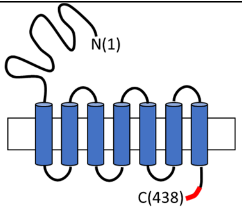	Whole serum	H, M	Abcam, Cambridge, UK No RRID
**SAB5500194 (SP235)**	Rabbit monoclonal IgG	aa 419-438 of $hVPAC_2$ LQFHRGSRAQSFLQTETSVI	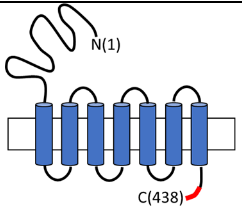	Protein A/G	H, R, M	Sigma-Aldrich, MO, USA No RRID
**SC-135604 (5B3)**	Mouse monoclonal IgG_{2a}	Internal region of $hVPAC_2$	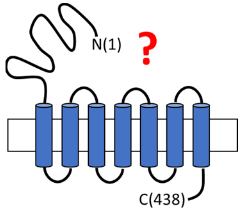	Unknown	H	Santa Cruz Biotechnology, CA, USA RRID: AB_2216692

H = human, R = rat, M = mouse. In brackets are species predicted to be reactive based on similarity in amino acid sequence.

Table S2. List of secondary antibodies.

Product	Product code	Source	Supplier, RRID
**Immunofluorescence**			
anti-mouse AlexaFluor 488+	A32723	Goat	Invitrogen, CA, USA RRID: AB_2633275
anti-mouse AlexaFluor 555+	A21127	Goat	Invitrogen, CA, USA RRID: AB_2535769
anti-mouse AlexaFluor 647+	A21236	Goat	Invitrogen, CA, USA RRID: AB_2535805
anti-rabbit AlexaFluor 488	A27034	Goat superclonal	Invitrogen, CA, USA RRID: AB_2536097
anti-rabbit AlexaFluor 555+	A32732	Goat	Invitrogen, CA, USA RRID: AB_2633281
anti-rabbit AlexaFluor 647+	A21245	Goat	Invitrogen, CA, USA RRID: AB_2535813
**Western blotting**			
anti-mouse HRP	A16072	Goat	Invitrogen, CA, USA RRID: AB_2534745
anti-rabbit HRP	A16110	Goat	Invitrogen, CA, USA RRID: AB_2534782

Secondary antibodies were used at a 1:1000 dilution for transfected Cos7 cells, 1:500 dilution for tissue and 1:10,000 dilution for western blotting experiments.

Table S3. Details of SD rat and post-mortem human TG cases used for immunofluorescence and western blotting

**Rat TG**				
**Rat**	**Sex**	**Age (weeks)**	**Weight**	**Histology/Western blotting**
#18	Male	16	533 g	Histology
#28	Female	17	311 g	Western blotting
#31	Female	18	277 g	Histology
#32	Male	18	478 g	Histology
#40	Male	17	593 g	Western blotting
#41	Male	17	442 g	Western blotting
**Human TG**				
**Case**	**Gender**	**Age (years)**	**Cause of death**	**Clinical notes**
3A	Female	92	Aspiration pneumonia/respiratory failure	Mild, slowly progressive dementia. No reported history of migraine
4A	Male	87	Myocardial infarction	No cognitive decline reported. No reported history of migraine
15A	Male	94	Bronchopneumonia	Slow, steady dementia. No reported history of migraine
1322	Male	16	Motor vehicle accident	None
1543*	Male	17	Motor vehicle accident	None

*TG = tissue from trigeminal nerve

Table S4. Protein amounts loaded for western blotting

**Antibody characterization western blots**	
pcDNA, human/rat PAC_1, human $VPAC_1$, human $VPAC_2$ membrane-enriched protein	1 µg and 10 µg
**Rat TG western blots**	
Rat TG membrane-enriched protein	10 µg
ab28670, ab183103, SC-377152, SAB5500193, SAB5500194 transfected Cos7 membrane-enriched protein	0.1 µg
SC-100315 transfected Cos7 membrane-enriched protein	1 µg
**Human TG western blots**	
Human TG	50 µg
ab28670, SC-377152, SAB5500193, SAB5500194 transfected Cos7 membrane-enriched protein	0.1 µg
ab183103, SC-100315 transfected Cos7 membrane-enriched protein	1 µg

Supporting Figures

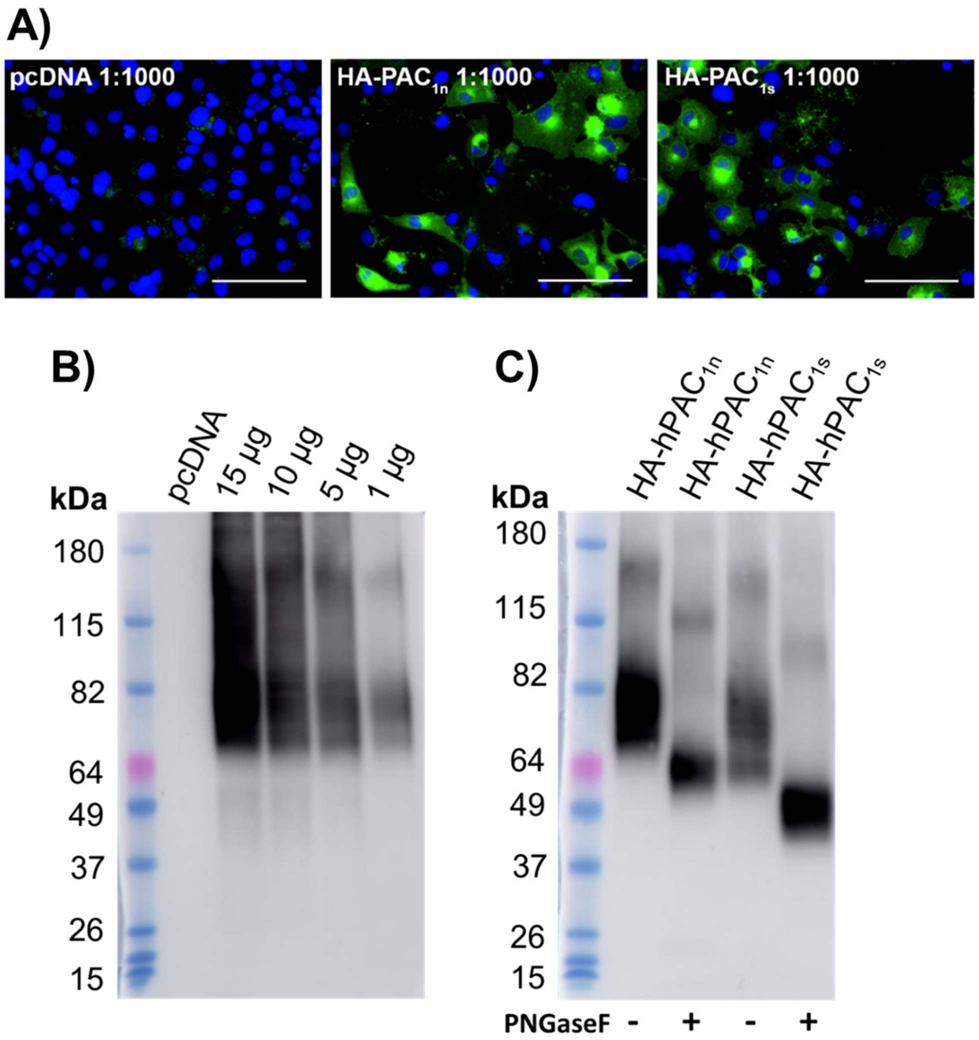

Figure S1. The anti-HA antibody successfully detected HA-tagged PAC_{1n} and PAC_{1s} receptors using fluorescent ICC and western blotting in transfected Cos7 cells. A) Cos7 cells were transfected with pcDNA or the HA-tagged PAC_{1n} and PAC_{1s} receptors and stained with anti-HA (1:1000). Immunoreactive staining is shown in green and nuclear DAPI staining in blue. Images were taken with a 40x non-confocal objective and represent one field of view from at least two experiments. Scale bar, 100 µm. B) Gradient of HA-PAC_{1n} protein quantities were used to determine the optimal protein loading amount. C) To provide reference for the potential range of PAC_1 receptor molecular masses that may be observed in tissues, glycosylated and deglycosylated PAC_1 receptor samples were compared. The PAC_1 receptor was deglycosylated using the PNGaseF enzyme, which removes the contribution of *N*-linked glycosylation. Western blots used protein samples prepared from Cos7 cells transfected with the B) HA-PAC_{1n} receptor or pcDNA, or C) HA-PAC_{1n} and HA-PAC_{1s} receptors (1 µg) treated with and without PNGaseF. The blots were probed with the anti-HA antibody (1:2000) and exposed for B) 1 second and C) 2 seconds. These images are representative of two independent experiments.

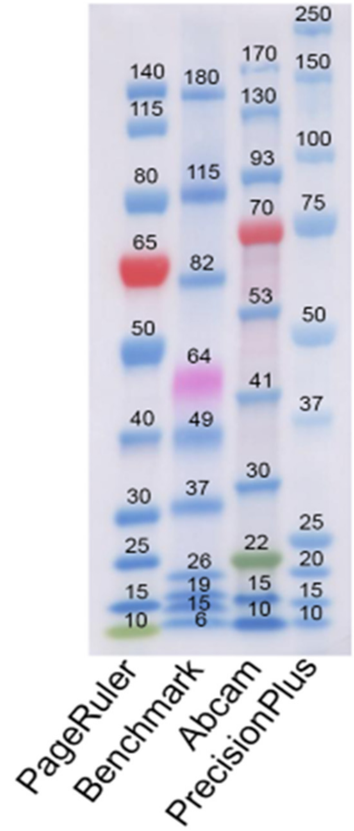

Figure S2. The benchmark protein ladder molecular markers run higher compared to other commercially available protein ladders under the conditions of this study. For comparison of protein ladders, the PageRuler (Life Technologies, Cat# 26616), Benchmark (Life Technologies, Cat# 10748010), Abcam (Abcam, Cat # ab116027) or Precision Plus (BIORAD, cat# 1610373) were used.

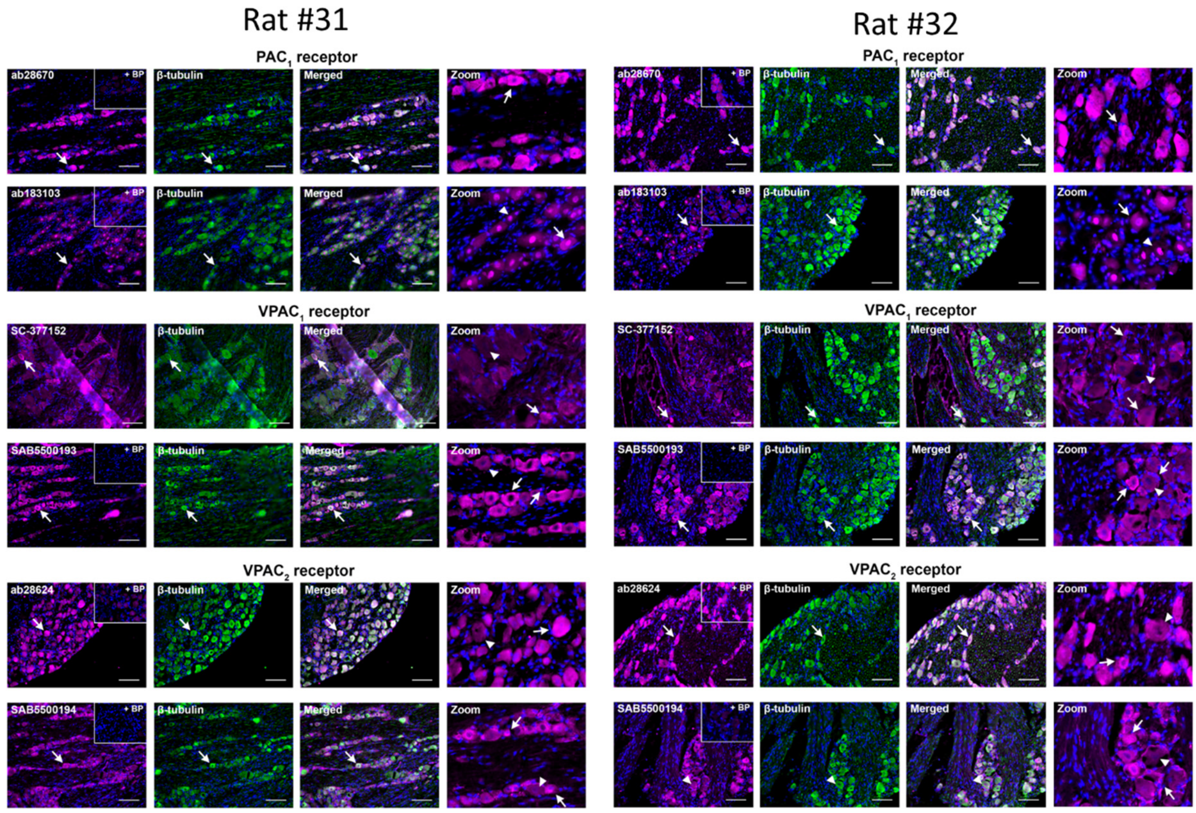

Figure S3. Independent experiments detecting PAC$_1$, VPAC$_1$, and VPAC$_2$ receptor-like immunoreactivity in rat trigeminal ganglia. PAC$_1$ (ab28670, 1:500; ab183103, 1:100), VPAC$_1$ (SC-377152, SAB5500193, 1:100) and VPAC$_2$ (ab28624, 1:500, SAB5500194, 1:100) receptor-like immunoreactivity in rat TG neurons and glia colocalized with β-tubulin (1:500). Pre-absorbed antibody controls are presented as insets. Receptor immunoreactivity is shown in pink, β-tubulin in green, colocalized regions appear white, and DAPI stained nuclei in blue. Arrows indicate examples of stained neurons or glia and arrowheads indicate examples of unstained cells. Images were taken with a 20x non-confocal objective. Scale bar, 100 µm. BP = blocking peptide.

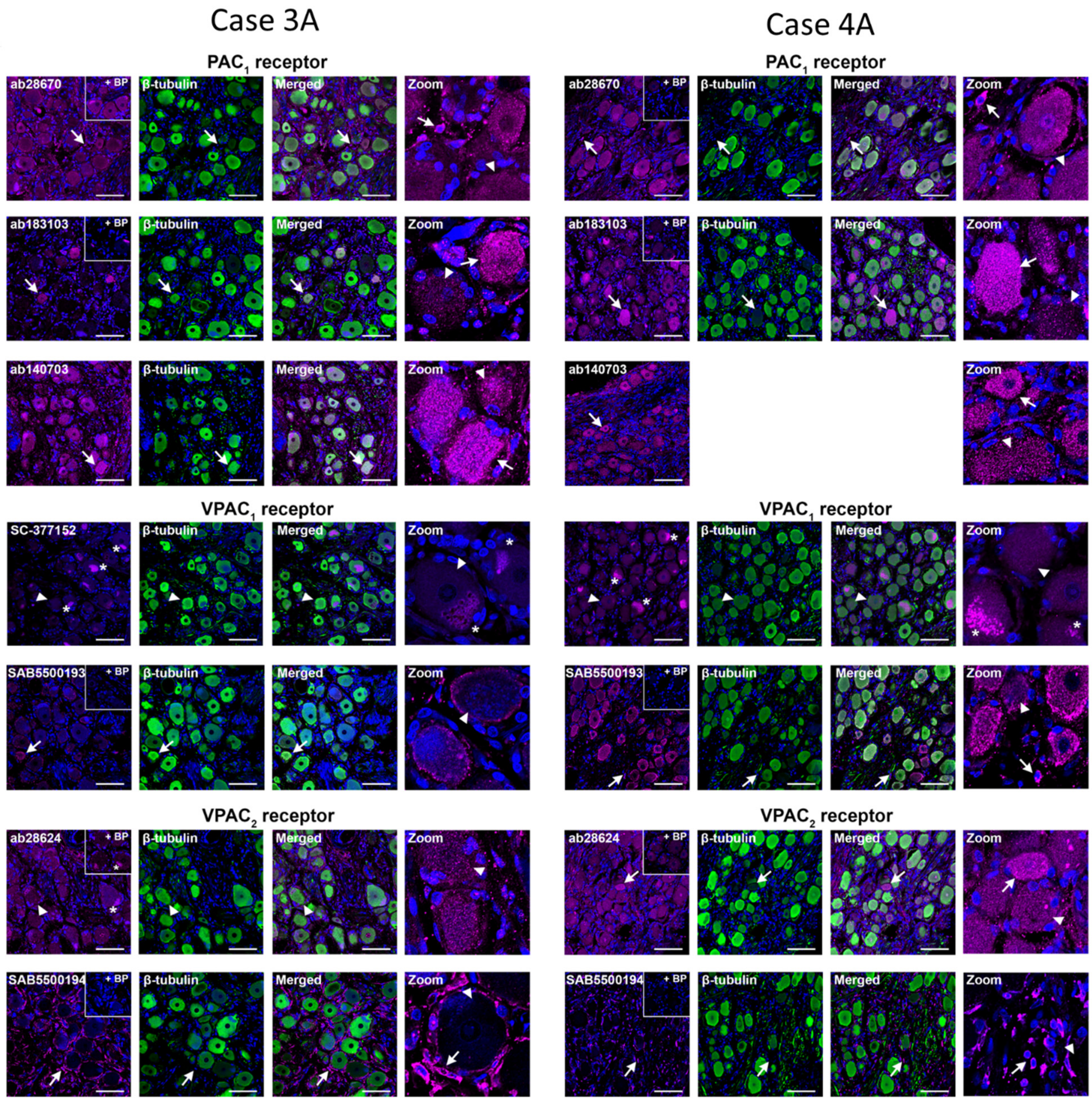

Figure S4. Independent experiments detecting PAC1, VPAC1, and VPAC2 receptor-like immunoreactivity in human trigeminal ganglia. PAC1 (ab28670, 1:500; ab183103, 1:100), VPAC1 (SC-377152, SAB5500193, 1:100) and VPAC2 (ab28624, 1:500, SAB5500194, 1:100) receptor-like immunoreactivity in human TG neurons and glia colocalized with β-tubulin (1:500). Pre-absorbed antibody controls are presented as insets. Receptor immunoreactivity is shown in pink, β-tubulin in green, colocalized regions appear white, and Hoechst stained nuclei in blue. ab140703 staining in case #4A was not co-stained with β-tubulin. Arrows indicate examples of stained neurons or glia and arrowheads indicate examples of unstained cells. Images were taken with a 20x confocal objective. Scale bar, 100 μm. BP = blocking peptide. *autofluorescence due to lipofuscin.

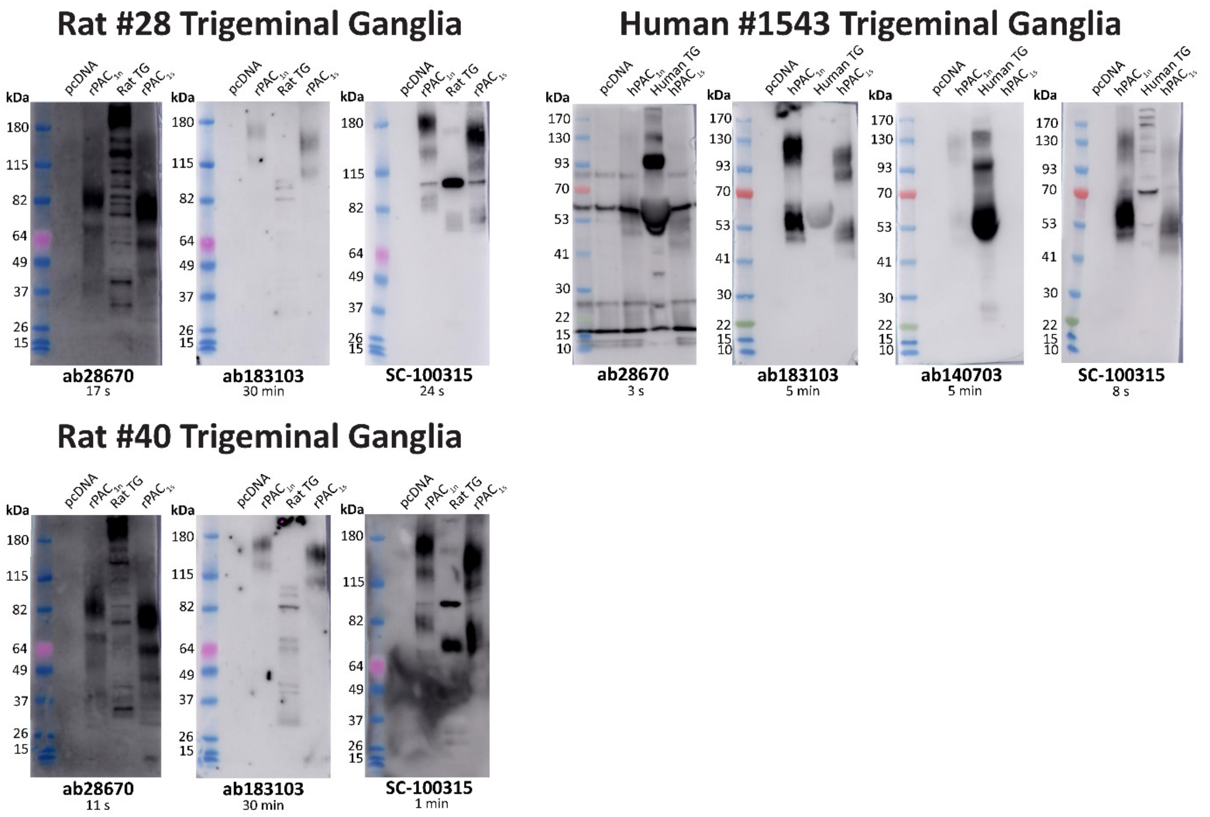

Figure S5. Western blotting independent experiments of PAC_1 receptor apparent molecular weights detected in rat and human trigeminal ganglia. Protein samples were prepared from rat TG (#28, 40) or human TG (case #1543) and Cos7 cells transfected with the rat or human PAC_{1n} and PAC_{1s} receptors or pcDNA. TG or Cos7 protein was loaded, and blots probed with anti-PAC_1 receptor antibodies (1:1000). Blots were exposed for the time indicated below each image.

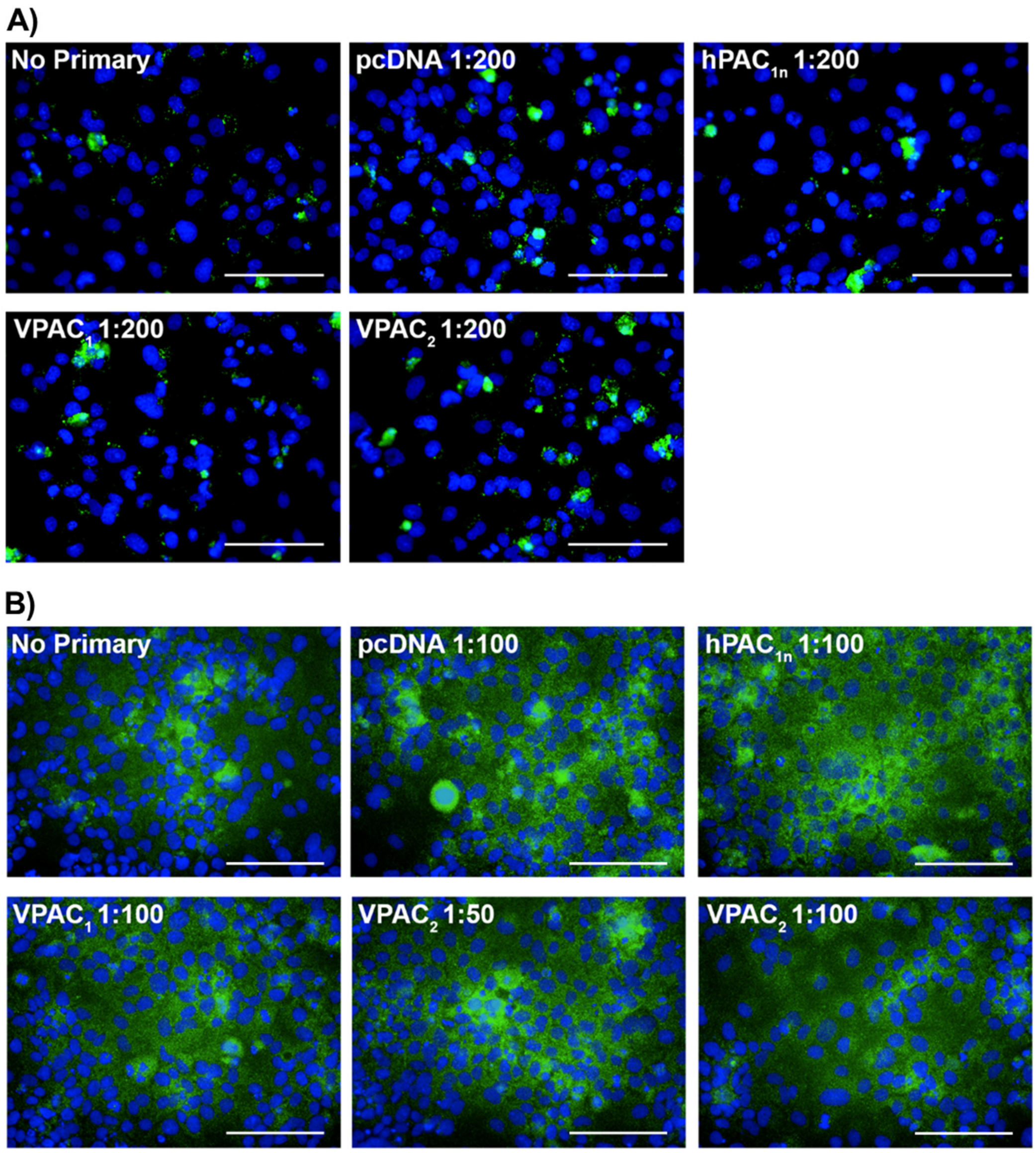

Figure S6. Lack of $VPAC_2$ receptor-like immunoreactivity by SC-135604 in transfected Cos7 cells. Cells were transfected with the $hPAC_{1n}$, $hVPAC_1$ and $hVPAC_2$ receptors or pcDNA. Cells were stained with SC-135604 (1:50-1:100) and subjected to A) no antigen retrieval or B) antigen retrieval. Antibody immunoreactivity is shown in green and nuclear DAPI staining in blue. The brightness of these images has been normalized to immunoreactivity observed in $VPAC_2$ receptor transfected cells with 1:100 antibody. Images were taken with a 40x non-confocal objective and represent one field of view from two independent experiments. Scale bar, 100 µm.

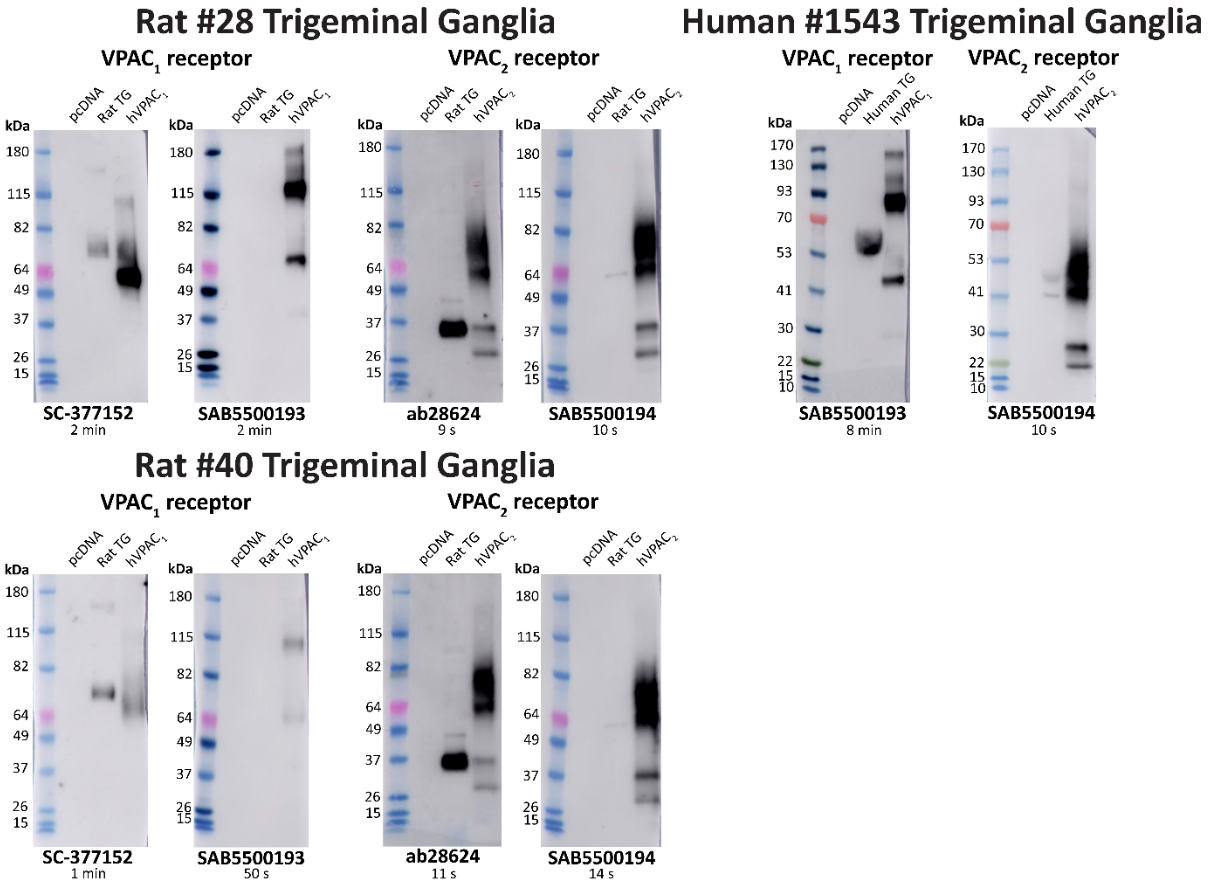

Figure S7. Western blotting independent experiments of $VPAC_1$ and $VPAC_2$ receptor apparent molecular weights detected in rat and human trigeminal ganglia. Protein samples were prepared from rat TG (#28, 40) or human TG (case #1543) and Cos7 cells transfected with the human $VPAC_1$ and $VPAC_2$ receptors or pcDNA. TG or Cos7 protein was loaded, and blots probed with anti-VPAC receptor antibodies (1:1000). Blots were exposed for the time indicated below each image.

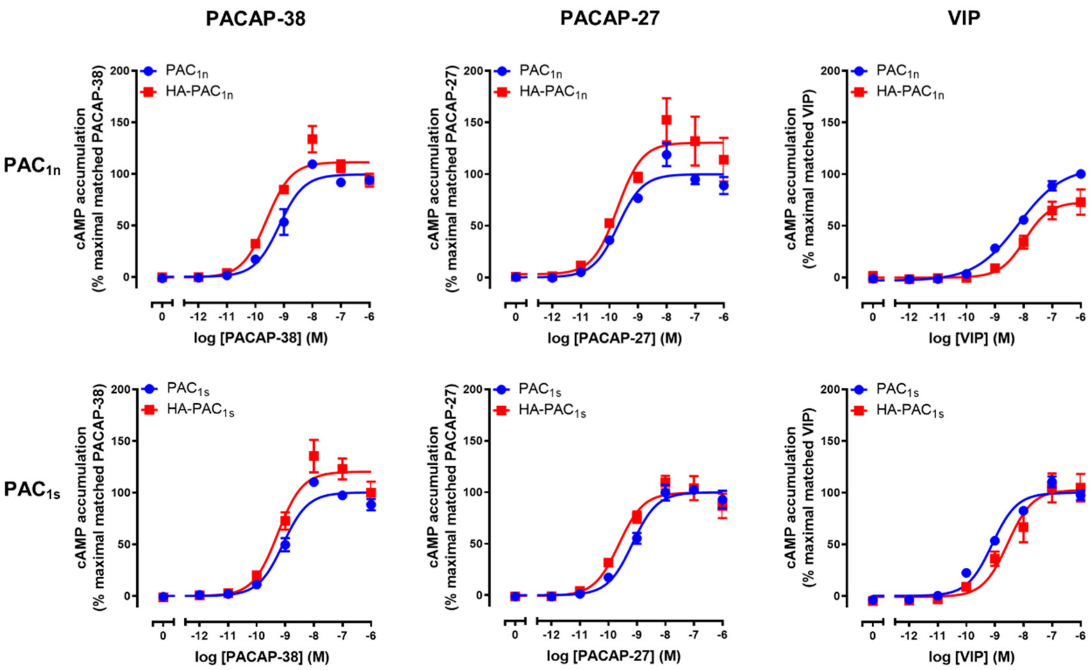

Figure S8. Comparison of agonist-stimulated cAMP accumulation at the un-tagged PAC_1 and HA-tagged PAC_1 receptors. Similar PACAP-38, PACAP-27 and VIP concentration-response curves were observed at the un-tagged and HA-tagged PAC_{1n} and HA-PAC_{1s} receptors. Data are expressed as the maximal cAMP accumulation observed by PACAP-38, PACAP-27 or VIP at the un-tagged receptor. Data points are the mean ± SEM of the combined data from three independent experiments.

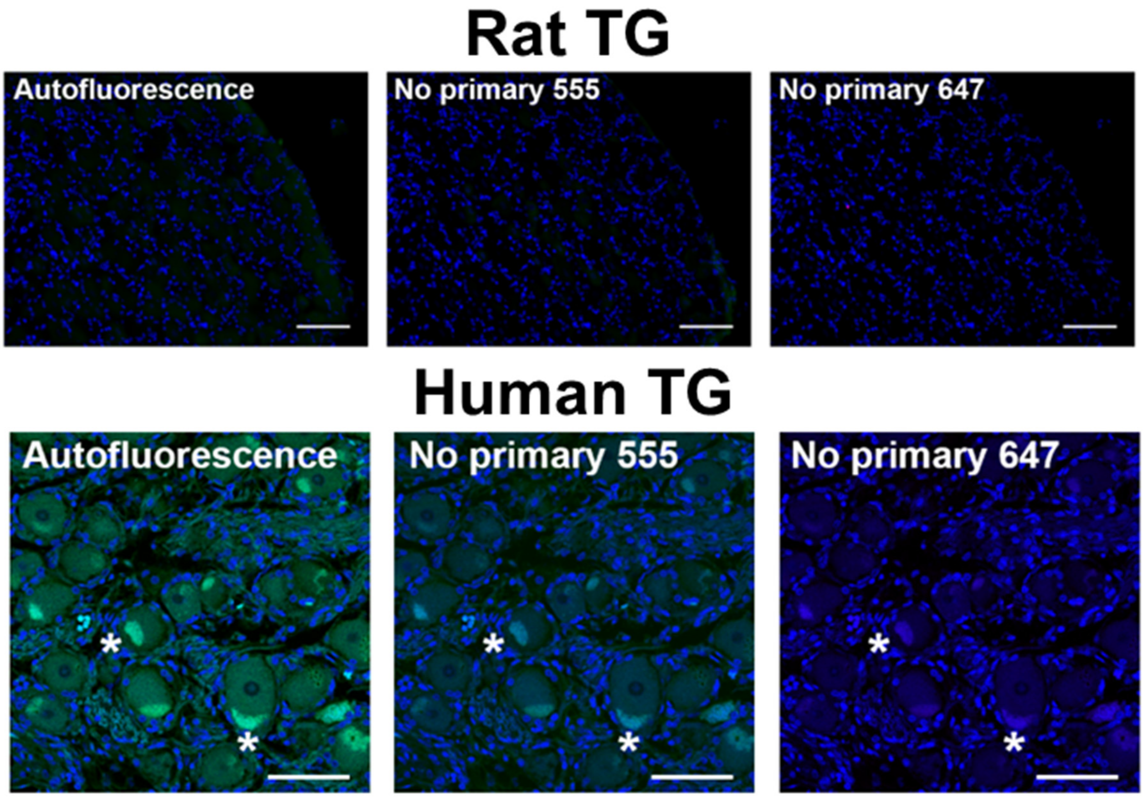

Figure S9. Autofluorescence and no primary controls of rat and human TG. Fluorescent signal indicating autofluorescence of the tissue was measured at 488 nm. Lack of fluorescent signal from the AlexaFluor555 and AlexaFluor647 secondary antibodies (1:500) in rat and human TG when the primary antibody was omitted. DAPI (rat) or Hoechst (human) was co-stained in all controls. Images were taken using a 20x non-confocal (rat) or confocal (human) objective and are representative of TG staining in three different cases. Scale bar, 100 µm. The brightness of these images was matched to their positively stained sections. *examples of autofluorescence due to lipofuscin.

Supporting Chemistry

Table SC1. Receptor antibody blocking peptide sequences and their purity.

Receptor antibody	Peptide antigen sequence	Purity
PAC_1 receptor ab28670	H_2N-LSKSSSQIRMSGLPADNLAT-NH_2	> 99%
PAC_1 receptor ab183103	H_2N-CLEKIQRANELMGF-$CONH_2$	> 99%
$VPAC_1$ receptor ab183312	H_2N-TRVSPGARRSSSFQAEVSLV-$CONH_2$	> 95%
$VPAC_2$ receptor ab28624	H_2N-LQFHRGSRAQSFLQTETSVI-COOH	> 99%
$VPAC_2$ receptor ab183334	H_2N-LQFHRGSRAQSFLQTETSVI-COOH	> 99%

General Procedure

All reagents were purchased as reagent grade and used without further purification. *N*,*N*-Diisopropylethylamine (DIPEA), piperidine, *N*,*N'*-diisopropylcarbodiimide (DIC), 1,2-ethanedithiol (EDT), triisopropylsilane (TIPS) and 4-methylmorpholine (NMM) were purchased from Sigma-Aldrich (St. Louis, Missouri). *O*-(7-Azabenzotriazol-1-yl)-*N*,*N*,*N'*,*N'*-tetramethyluronium hexafluorophosphate (HATU), Fmoc-Ala-OH, Fmoc-Arg(Pbf)-OH (Pbf = 2,2,4,6,7-pentamethyIdlhydrobenzofuran-5-sulfonyl), Fmoc-Asn(Trt)-OH (Trt = triphenylmethane), Fmoc-Asp(*t*Bu)-OH (*t*Bu = *tert*-butyl), Fmoc-Cys(Trt)-OH, Fmoc-Glu(*t*Bu)-OH, Fmoc-Gln(Trt)-OH, Fmoc-Gly-OH, Fmoc-His(Trt)-OH, Fmoc-lle-OH, Fmoc-Leu-OH, Fmoc-Lys(Boc)-OH (Boc = *tert*-butoxycarbonyl), Fmoc-Met-OH, Fmoc-Phe-OH, Fmoc-Pro-OH, Fmoc-Ser(*t*Bu)-OH, Fmoc-Thr(*t*Bu)-OH, Fmoc-Val-OH and 4-[(2,4-Dimethoxyphenyl)(Fmoc-amino)methyl]phenoxyacetic acid (Rink amide linker) were purchased from CS Bio (Shanghai, China). 4-(Dimethylamino)pyridine (DMAP) was purchased from Novabiochem (Darmstadt, Germany). 6-Chloro-1-hydroxybenzotriazole (6-Cl-HOBt) was purchased from Apptec (Louisville, Kentucky). Aminomethyl polystyrene resin was purchased from Rapp Polymere (Tübingen, Germany). 4-(Hydroxymethyl)phenoxyacetic acid (HMP linker) was purchased from AK Scientific (Union City, California). Yields refer to chromatographically homogeneous materials. Microwave reactions were carried out on a Biotage® Initiator+ Alstra™ (Uppsala, Sweden) automated peptide synthesizer. Semi-preparative/analytical RP-HPLC was performed on a Thermo Scientific (Waltham, MA) Dionex Ultimate 3000 HPLC equipped with a four channel UV detector at 210, 225, 254 and 280 nm using either an analytical column (Waters (Milford, MA) XTerra® MS C18, (5 µm; 4.6 × 150 mm) at a flow rate of 1 mL min^{-1}, or a Phenomenex® semi-preparative column (Torrance, CA), Gemini C18, (5 µm; 10 × 250 mm) at a flow rate of 4 mL min^{-1}. A suitably adjusted gradient of 5% B to 95% B was used, where solvent A was 0.1% TFA in H_2O and B was 0.1% TFA in acetonitrile. LC-MS spectra were acquired using Agilent Technologies (Santa Clara, CA) 1260 Infinity LC equipped with an Agilent Technologies 6120 Quadrupole mass spectrometer. An analytical column (Agilent ZORBAX 300SB-C3, 3.5 µm; 3.0 × 150 mm) was used at a flow rate of 0.3 mL min^{-1} using a linear gradient of 5% B to 95% B over 30 min, where solvent A was 0.1% formic acid in H_2O and B was 0.1% formic acid in acetonitrile.

General Methods

Method 1: General procedure for attachment of Fmoc Rink amide to the resin:

To aminomethyl polystyrene resin (80 mg, 0.1 mmol, loading: 1.26 mmol/g) pre-swollen in CH_2Cl_2 (5 mL, 20 min), was added 4-[(2,4-dimethoxyphenyl)(Fmoc-amino)methyl]phenoxyacetic acid (220.2 mg, 4 equiv., 0.4 mmol) and 6-Cl-HOBt (70 mg, 3.5 equiv., 0.35 mmol) dissolved in DMF (1.5 mL) followed by addition of DIC (62 µL, 4 equiv., 0.4 mmol). The reaction mixture was gently agitated at room temperature for 24 h. The resin was filtered and washed with DMF (3 × 3 mL) after which a negative ninhydrin test confirmed successful coupling.

Method 2: General procedure for attachment of HMP to the resin:

To aminomethyl polystyrene resin (80 mg, 0.1 mmol, loading: 1.26 mmol/g) pre-swollen in CH_2Cl_2 (5 mL, 20 min), was added 4-(hydroxymethyl)phenoxyacetic acid (80 mg, 4 equiv., 0.4 mmol) and 6-Cl-HOBt (70 mg, 3.5 equiv., 0.35 mmol) dissolved in DMF (1.5 mL) followed by addition of DIC (62 µL, 4 equiv., 0.4 mmol). The reaction mixture was gently agitated at room temperature for 24 h. The resin was filtered and washed with DMF (3 × 3 mL) after which a negative ninhydrin test confirmed successful coupling.

Method 3: General procedure for esterification between HMP and amino acid:

To HMP resin (0.1 mmol) pre-swollen in CH_2Cl_2 (5 mL, 20 min), was added Fmoc-Ile-OH (70 mg, 2 equiv., 0.2 mmol) and DMAP (catalytic amount) dissolved in DMF (1 mL) followed by addition of DIC (32 µL, 2 equiv., 0.2 mmol). The reaction mixture was gently agitated at room temperature for 1 h. The resin was filtered and washed with DMF (3 × 3 mL) and repeated with (2 × 1 h) coupling cycles with fresh reagents.

Method 4: General procedure for removal of N^{α}-Fmoc-protecting group:

Peptidyl resin was treated with a solution of 20 vol % piperidine in DMF (*v*/*v*, 4 mL) and the mixture was agitated on the Biotage® Initiator Alstra for 2 × 5 min at room temperature. The resin was filtered and washed with DMF (3 × 3 mL).

Method 5: General coupling procedure for Fmoc-Ala-OH, Fmoc-Asn(Trt)-OH, Fmoc-Asp(*t*Bu)-OH, Fmoc-Gln(Trt)-OH, Fmoc-Glu(*t*Bu)-OH Fmoc-Gly-OH, Fmoc-Ile-OH, Fmoc-Leu-OH, Fmoc-Lys(Boc)-OH, Fmoc-Met-OH, Fmoc-Phe-OH, Fmoc-Pro-OH, Fmoc-Ser(*t*Bu)-OH, Fmoc-Thr(*t*Bu)-OH, Fmoc-Tyr(*t*Bu)-OH, Fmoc-Val-OH:

Couplings were performed using the Biotage® Initiator Alstra with the appropriate Fmoc-protected amino acid (0.2 M, DMF, 5 equiv.), HATU (0.5 M, DMF, 4.75 equiv.) and NMM in DMF (2 M, 8 equiv.) using a single coupling cycle at 75 °C, 110 W for 5 min. The resin was filtered and washed with DMF (3 × 3 mL).

Method 6: General coupling procedure for Fmoc-Arg(Pbf)-OH:

Double coupling cycles of Fmoc-Arg(Pbf)-OH were carried out with the Biotage® Initiator Alstra using Fmoc-Arg(Pbf)-OH (0.2 M, DMF, 5 equiv.), HATU (0.5 M, DMF, 4.75 equiv.) and NMM in DMF (2 M, 8 equiv.) with first coupling at room temperature for 25 min, followed by a second coupling cycle using fresh reagents at 72 °C, 110 W for 5 min. The resin was filtered and washed with DMF (3 × 3 mL).

Method 7: General coupling procedure for Fmoc-Cys(Trt)-OH and Fmoc-His(Trt)-OH:

Double coupling cycles of Fmoc-Cys(Trt)-OH and Fmoc-His(Trt)-OH were carried out with the Biotage® Initiator Alstra using Fmoc protected amino acid (0.2 M, DMF, 5 equiv.), HATU (0.5 M, DMF, 4.75 equiv.) and NMM in DMF (2 M, 8 equiv.) with first coupling at room temperature for 15 min, followed by a second coupling using fresh reagents at 43 °C, 110 W for 10 min. The resin was filtered and washed with DMF (3 × 3 mL).

Method 8: General procedure for the capping of free amino groups:

Fmoc-protected peptidyl resin was treated with 5 M Ac_2O in DMF (0.47 mL, 2.5 equiv.) and NMM in DMF (2 M, 8 equiv.) using the Biotage® Initiator Alstra at room temperature for 10 min. The resin was filtered and washed with DMF (3 × 3 mL).

Method 9: General procedure for TFA-mediated resin cleavage and global deprotection:

Peptidyl resin was treated with a mixture of TFA/H_2O/TIPS/EDT (94:2.5:2.5:1, *v/v/v/v*, 10 mL) for 120 min. The filtrate was partially concentrated under a gentle stream of N_2, then cold diethyl ether was then added to form a precipitate. The mixture was centrifuged, and the solution was carefully decanted off and discarded, before dissolving the solid pellet in H_2O:acetonitrile (1:1, *v/v,* 25 mL) containing 0.1% TFA and lyophilised.

Synthesis of anti-PAC1 receptor antibody (ab28670) blocking peptide using Fmoc-SPPS.

Scheme SC1. PAC1 (ab28670) blocking peptide.

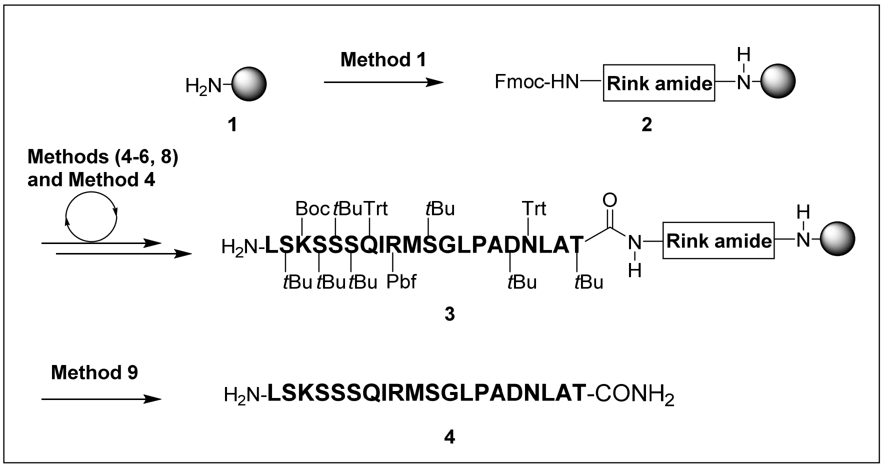

Synthesis of PAC1 (ab28670) blocking peptide (**4**).

Fmoc-Rink amide was attached to aminomethyl polystyrene resin **1** using **Method 1** followed by Fmoc-removal using **Method 4**. Direct attachment of Fmoc-Thr(*t*Bu)-OH at position 20 to resin bound Rink amide **2** was achieved using **Method 5**. **Method 4** was used for all subsequent N^{α}-Fmoc removals where appropriate. Linear elongation of the peptide chain was achieved by coupling appropriate Fmoc-amino acids up to ^{1}Leu indicated in **Scheme SC1** using **Method 5**. All Fmoc-Arg(Pbf)-OH residues are coupled using **Method 6**. Capping of free amino groups was conducted throughout the synthesis using **Method 8**. Peptide **3** was liberated from resin using **Method 9** affording **4**. The crude **4** reaction mixture was purified batchwise by semi-preparative RP-HPLC on a Phenomenex® Gemini C18 column (10 × 250 mm, 5 µm) using a linear gradient of 5% to 95% over 95 min (*ca.* 1% B/min) with a flow rate of 4 mL/min. Fractions were collected at 0.2 min intervals and analysed by ESI-MS and RP-HPLC. Fractions identified with correct *m*/*z* were combined and lyophilised to afford the *title compound* **4** as a white amorphous solid (66 mg, 32% yield based on 0.1 mmol scale), t_R = 10.1 min, > 98% purity by HPLC; (**Figure SC1** and **SC2**).

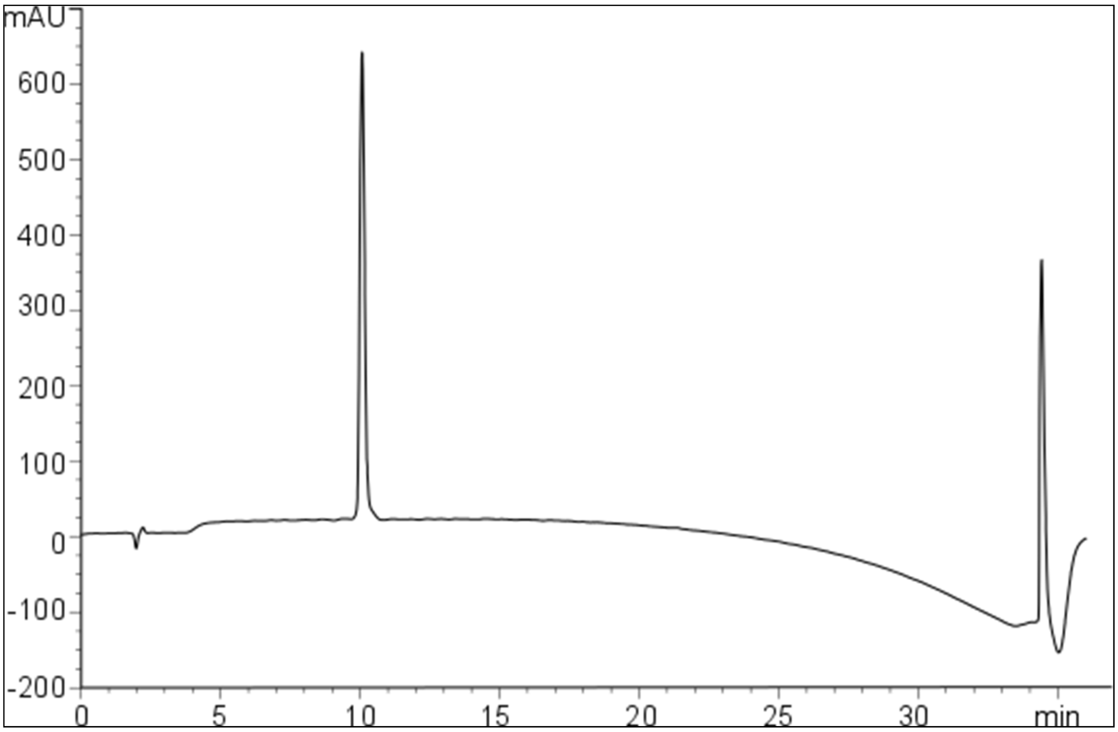

Figure SC1: Analytical RP-HPLC chromatogram of purified peptide **4**, t_R = 10.1 min. Chromatographic separations were performed on a Thermo Scientific Dionex Ultimate 3000 HPLC using a XTerra® MS C-18 column (5 µm; 4.6 × 150 mm) and a linear gradient of 5-95% B in 35 min at room temperature, *ca*. 3% B per min at a flow rate of 1.0 mL/min. Buffer A: H_2O containing 0.1% TFA (*v/v*); Buffer B: acetonitrile containing 0.1% TFA (*v/v*).

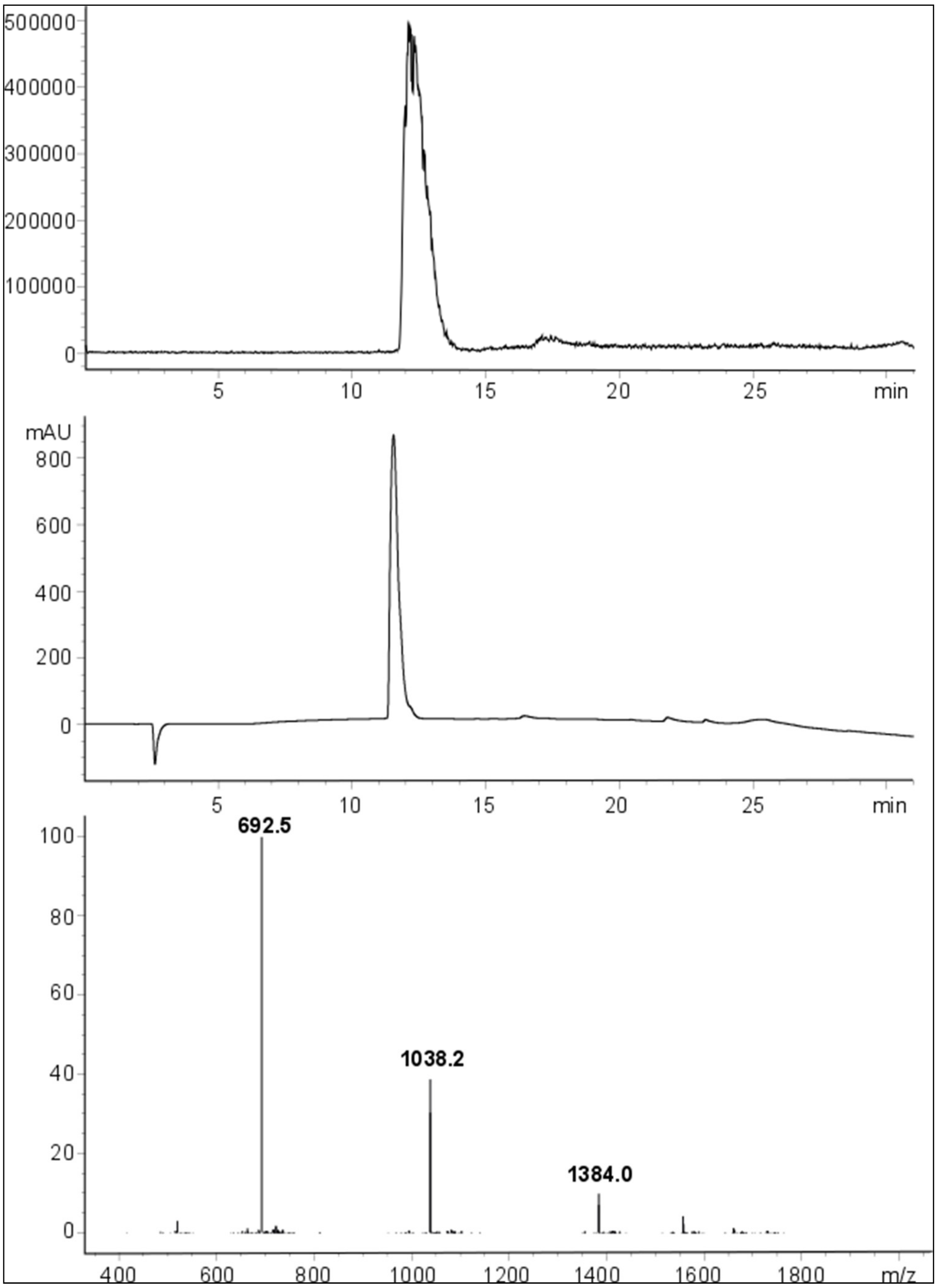

Figure SC2: LC-MS profile of purified peptide **4**; ion polarity positive operating at a nominal accelerating voltage of 70 eV. ESI-MS. (*m/z* $[M+2H]^{2+}$ calcd: 1038.2; found: 1038.2; $[M+3H]^{3+}$ calcd: 692.7; found: 692.5; $[2M+3H]^{3+}$ calcd: 1384.5; found: 1384.0. Mass deconvolution calculated at 2074.45 Da with standard deviation of 0.07; theoretical mass calculated at 2075.37 Da.

Synthesis of anti-PAC1 receptor antibody (ab183103) blocking peptide using Fmoc-SPPS.

Scheme SC2. PAC1 (ab183103) blocking peptide.

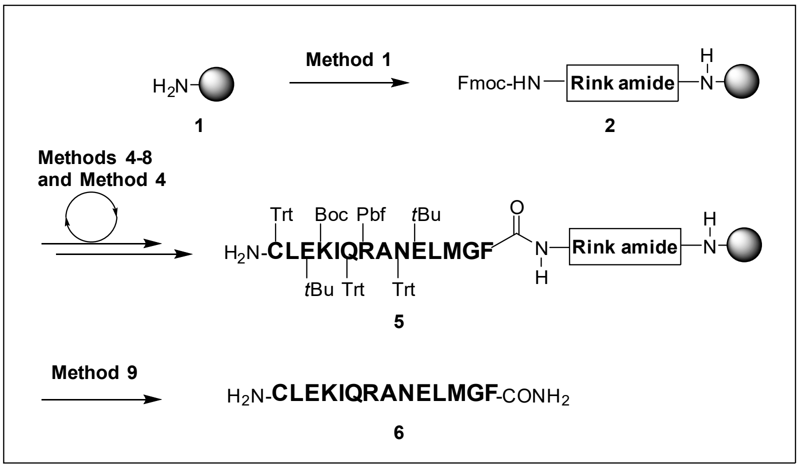

Synthesis of PAC1 (ab183103) blocking peptide (**6**).

Fmoc-Rink amide was attached to aminomethyl polystyrene resin **1** using **Method 1** followed by Fmoc-removal using **Method 4**. Direct attachment of Fmoc-Phe-OH at position 14 to resin bound Rink amide **2** was achieved using **Method 5**. **Method 4** was used for all subsequent N^{α}-Fmoc removals where appropriate. Linear elongation of the peptide chain was achieved by coupling appropriate Fmoc-amino acids up to ^{1}Cys indicated in **Scheme SC2** using **Method 5**. All Fmoc-Arg(Pbf)-OH residues are coupled using **Method 6**. All Fmoc-Cys(Trt)-OH residues are coupled using **Method 7**. Capping of free amino groups was conducted throughout the synthesis using **Method 8**. Peptide **5** was liberated from resin using **Method 9** affording **6**. The crude **6** reaction mixture was purified batchwise by semi-preparative RP-HPLC on a Phenomenex® Gemini C18 column (10 × 250 mm, 5 µm) using a linear gradient of 5% to 95% over 95 min (*ca.* 1% B/min) with a flow rate of 4 mL/min. Fractions were collected at 0.2 min intervals and analysed by ESI-MS and RP-HPLC. Fractions identified with correct *m*/*z* were combined and lyophilised to afford the *title compound* **6** as a white amorphous solid (34 mg, 21% yield based on 0.1 mmol scale), t_R = 21.8 min, > 99% purity by HPLC; (**Figure SC3** and **SC4**).

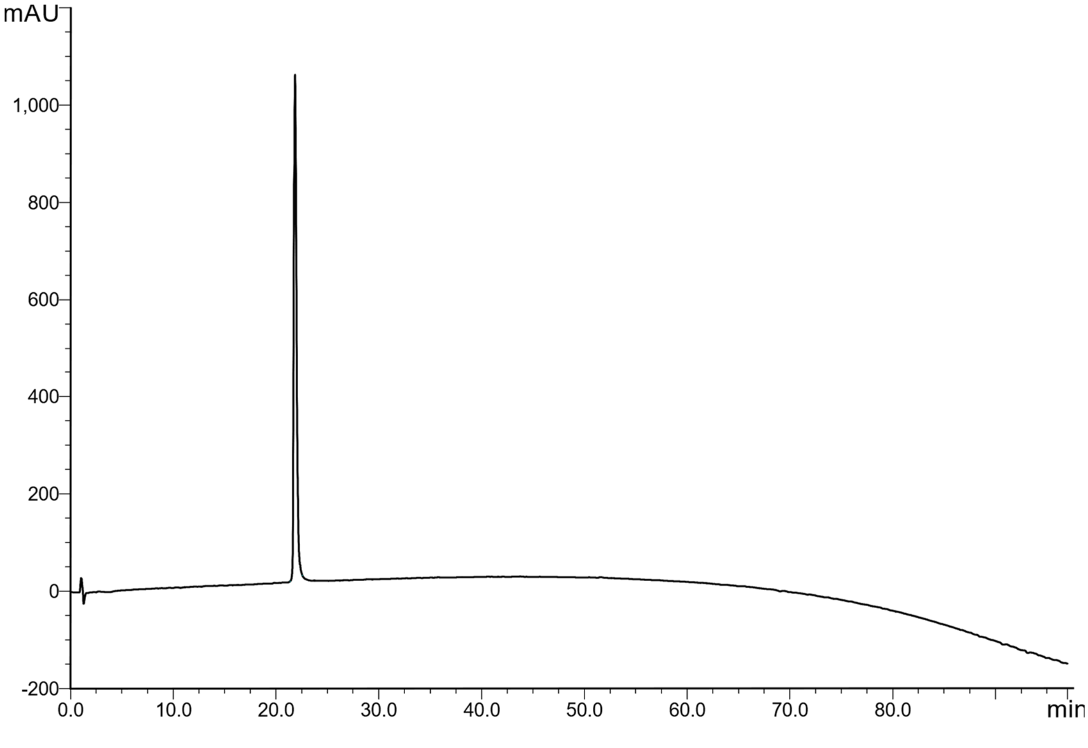

Figure SC3: Analytical RP-HPLC chromatogram of purified peptide **6**, t_R = 21.8 min. Chromatographic separations were performed on a Thermo Scientific Dionex Ultimate 3000 HPLC using a XTerra® MS C-18 column (5 µm; 4.6 × 150 mm) and a linear gradient of 5-95% B in 95 min at room temperature, *ca*. 1% B per min at a flow rate of 1.0 mL/min. Buffer A: H_2O containing 0.1% TFA (*v/v*); Buffer B: acetonitrile containing 0.1% TFA (*v/v*).

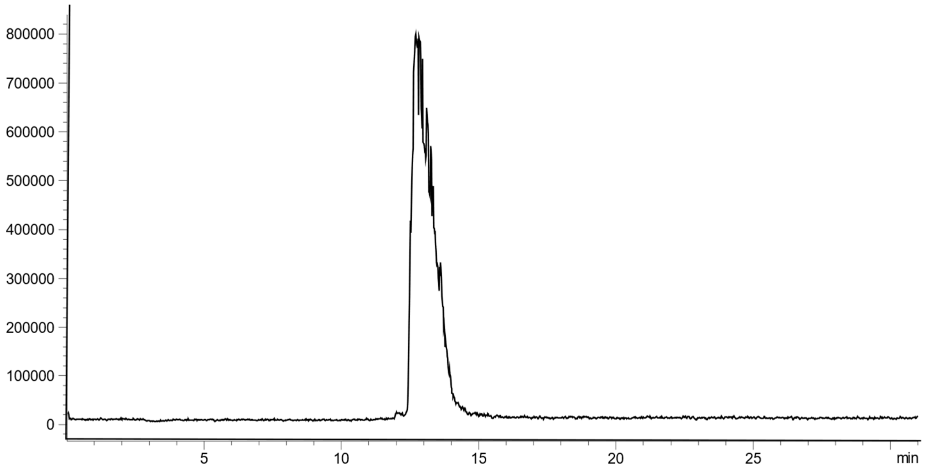

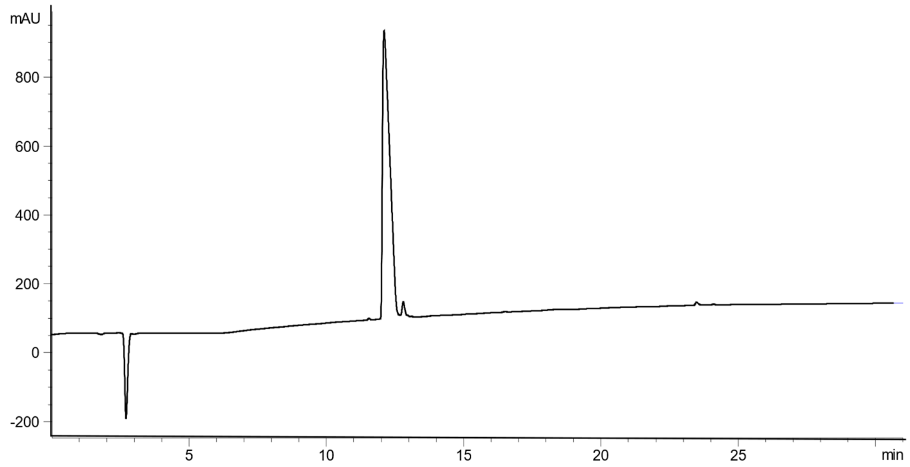

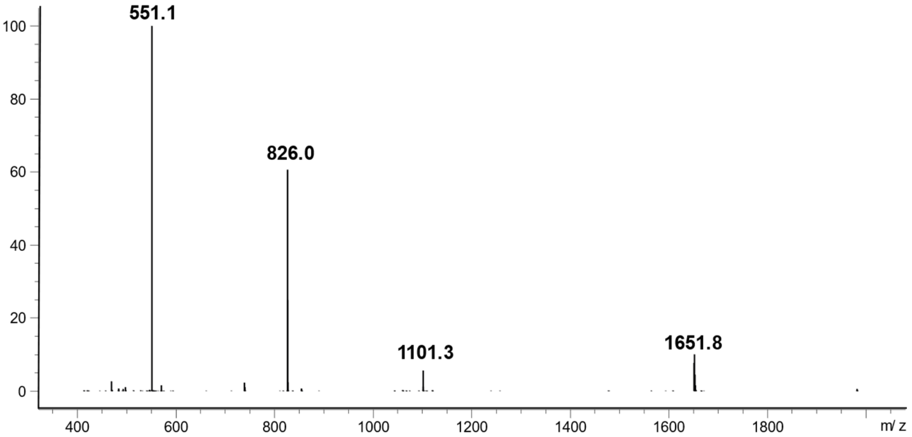

Figure SC4: LC-MS profile of purified peptide **6**; ion polarity positive operating at a nominal accelerating voltage of 70 eV. ESI-MS (*m/z* $[M+1H]^{1+}$ calcd: 1651.5; found: 1651.8; $[M+2H]^{2+}$ calcd: 826.2; found: 826.0; $[M+3H]^{3+}$ calcd: 551.2; found: 551.1; $[2M+3H]^{3+}$ calcd: 1101.3; found: 1101.3. Mass deconvolution calculated at 1650.37 Da with standard deviation of 0.40; theoretical mass calculated at 1650.45 Da.

Synthesis of anti-VPAC2 receptor antibody (ab28624) blocking peptide using Fmoc-SPPS.

Scheme SC3. **V**PAC2 (ab28624) blocking peptide.

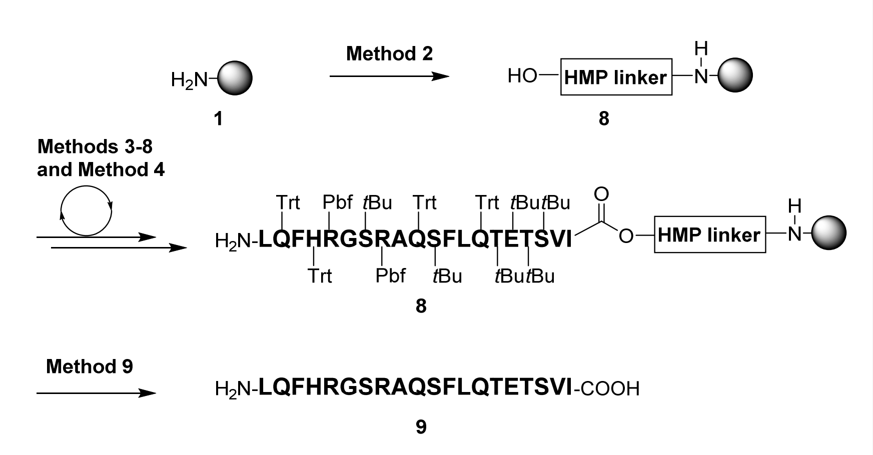

<u>Synthesis of VPAC2 (ab28624) blocking peptide (**9**)</u>.

HMP linker was attached to aminomethyl polystyrene resin **1** using **Method 2**. Direct attachment of Fmoc-Ile-OH at position 20 to resin bound HMP **8** was achieved using **Method 3**. **Method 4** was used for all subsequent N^{α}-Fmoc removals where appropriate. Linear elongation of the peptide chain was achieved by coupling appropriate Fmoc-amino acids up to ^{1}Leu indicated in **Scheme SC3** using **Method 5**. All Fmoc-Arg(Pbf)-OH residues are coupled using **Method 6**. All Fmoc-His(Trt)-OH residues are coupled using **Method 7**. Capping of free amino groups was conducted throughout the synthesis using **Method 8**. Peptide **8** was liberated from resin using **Method 9** affording **9**. The crude **9** reaction mixture was purified batchwise by semi-preparative RP-HPLC on a Phenomenex® Gemini C18 column (10 × 250 mm, 5 μm) using a linear gradient of 5% to 95% over 95 min (*ca.* 1% B/min) with a flow rate of 4 mL/min. Fractions were collected at 0.2 min intervals and analysed by ESI-MS and RP-HPLC. Fractions identified with correct *m*/*z* were combined and lyophilised to afford the *title compound* **9** as a white amorphous solid (15 mg, 6% yield based on 0.1 mmol scale), t_R = 23.6 min, > 99% purity by HPLC; (**Figure SC5** and **SC6**).

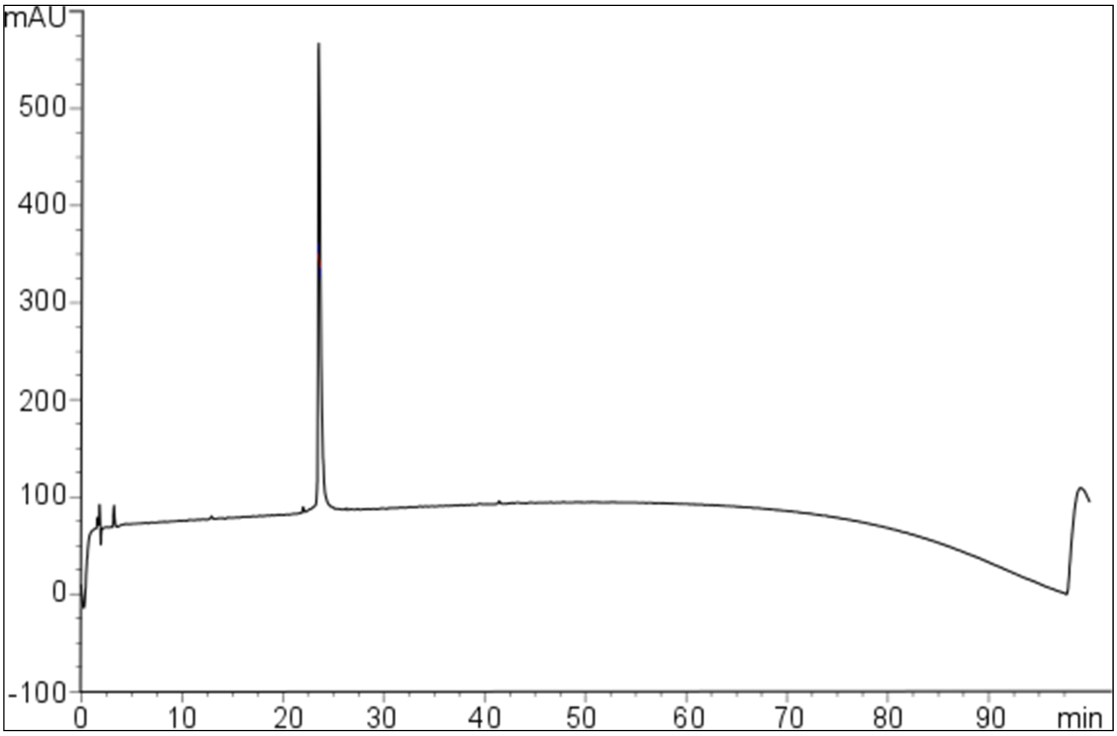

Figure SC5: Analytical RP-HPLC chromatogram of purified peptide **9**, t_R = 23.6 min. Chromatographic separations were performed on a Thermo Scientific Dionex Ultimate 3000 HPLC using a XTerra® MS C-18 column (5 µm; 4.6 × 150 mm) and a linear gradient of 5-95% B in 95 min at room temperature, *ca*. 1% B per min at a flow rate of 1.0 mL/min. Buffer A: H_2O containing 0.1% TFA (*v/v*); Buffer B: acetonitrile containing 0.1% TFA (*v/v*).

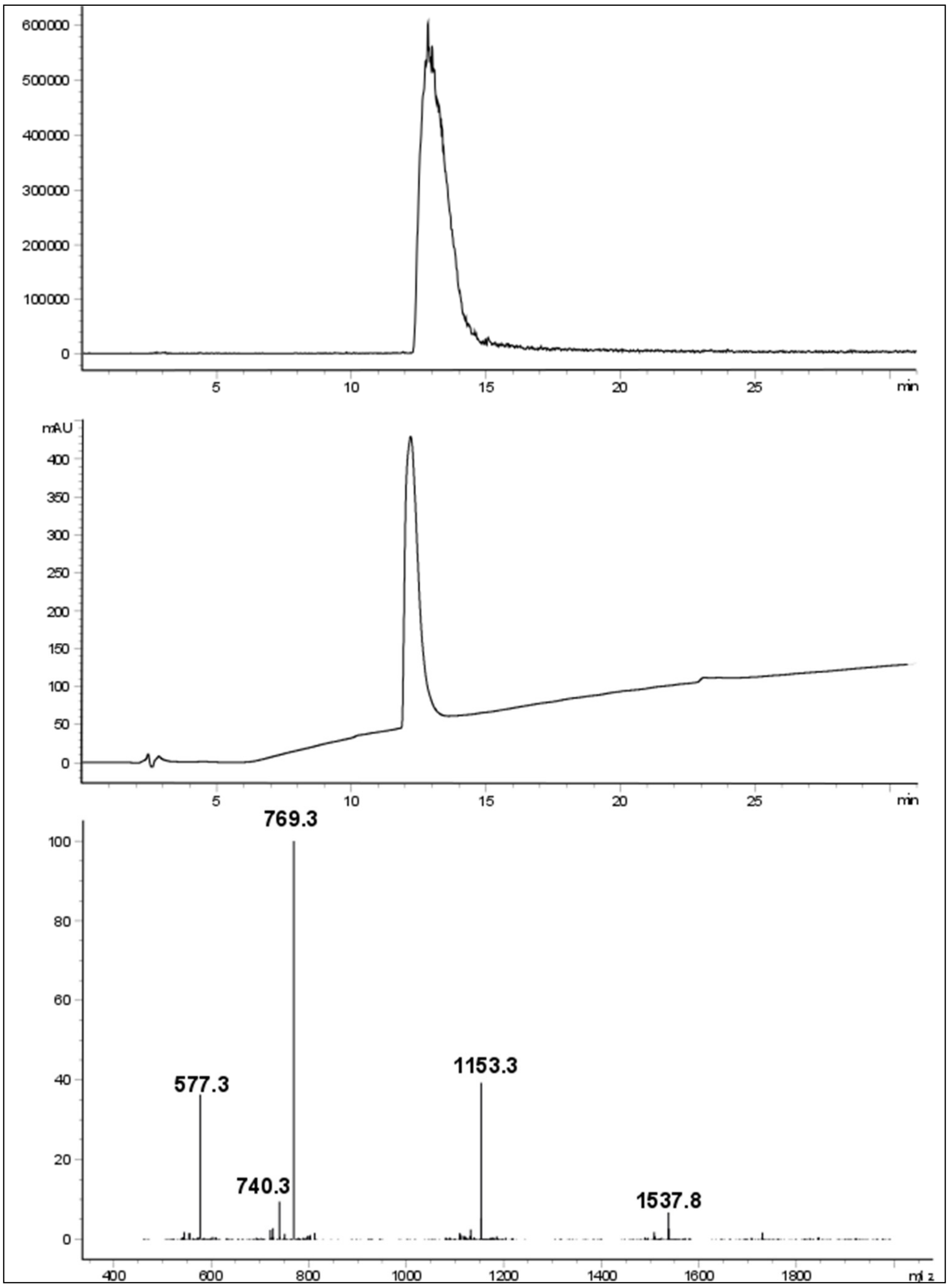

Figure SC6: LC-MS profile of purified peptide **9**; ion polarity positive operating at a nominal accelerating voltage of 70 eV. ESI-MS (*m/z* $[M+2H]^{2+}$ calcd: 1153.8; found: 1153.3; $[M+3H]^{3+}$ calcd: 769.5; found: 769.3; $[M+4H]^{4+}$ calcd: 577.4; found: 577.3; $[2M+3H]^{3+}$ calcd: 1538.0; found: 1537.8. Mass deconvolution calculated at 2304.9 Da with standard deviation of 0.30; theoretical mass calculated at 2305.59 Da.

Synthesis of anti-VPAC1 receptor antibody (SAB5500193) blocking peptide using Fmoc-SPPS.

Scheme SC4. VPAC1 (SAB5500193) blocking peptide.

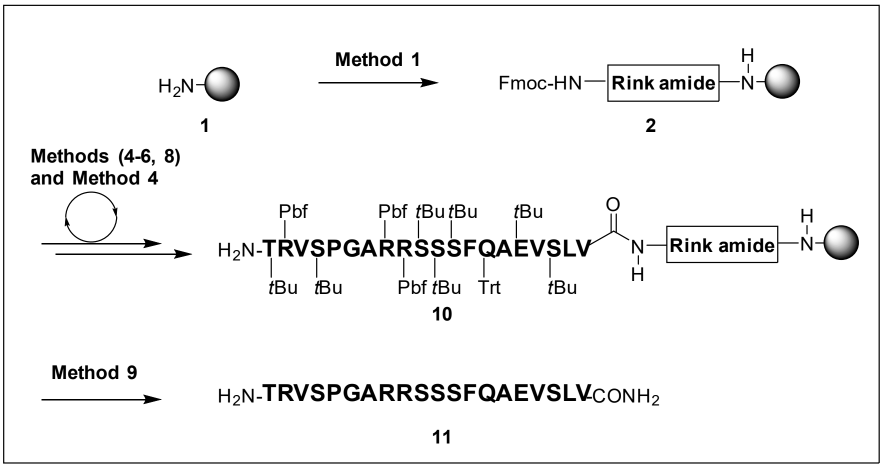

Synthesis of VPAC1 (SAB5500193) blocking peptide (**11**).

Fmoc-Rink amide was attached to aminomethyl polystyrene resin **1** using **Method 1** followed by Fmoc-removal using **Method 4**. Direct attachment of Fmoc-Val-OH at position 20 to resin bound Rink amide **2** was achieved using **Method 5**. **Method 4** was used for all subsequent N^{α}-Fmoc removals where appropriate. Linear elongation of the peptide chain was achieved by coupling appropriate Fmoc-amino acids up to ^{1}Thr indicated in **Scheme SC4** using **Method 5**. All Fmoc-Arg(Pbf)-OH residues are coupled using **Method 6**. Capping of free amino groups was conducted throughout the synthesis using **Method 8**. Peptide **10** was liberated from resin using **Method 9** affording **11**. The crude **11** reaction mixture was purified batchwise by semi-preparative RP-HPLC on a Phenomenex® Gemini C18 column (10 × 250 mm, 5 µm) using a linear gradient of 5% to 95% over 95 min (*ca.* 1% B/min) with a flow rate of 4 mL/min. Fractions were collected at 0.2 min intervals and analysed by ESI-MS and RP-HPLC. Fractions identified with correct *m*/*z* were combined and lyophilised to afford the *title compound* **11** as a white amorphous solid (2 mg, 1% yield based on 0.1 mmol scale), t_R = 18.2 min, > 95% purity by HPLC; (**Figure SC7** and **SC8**).

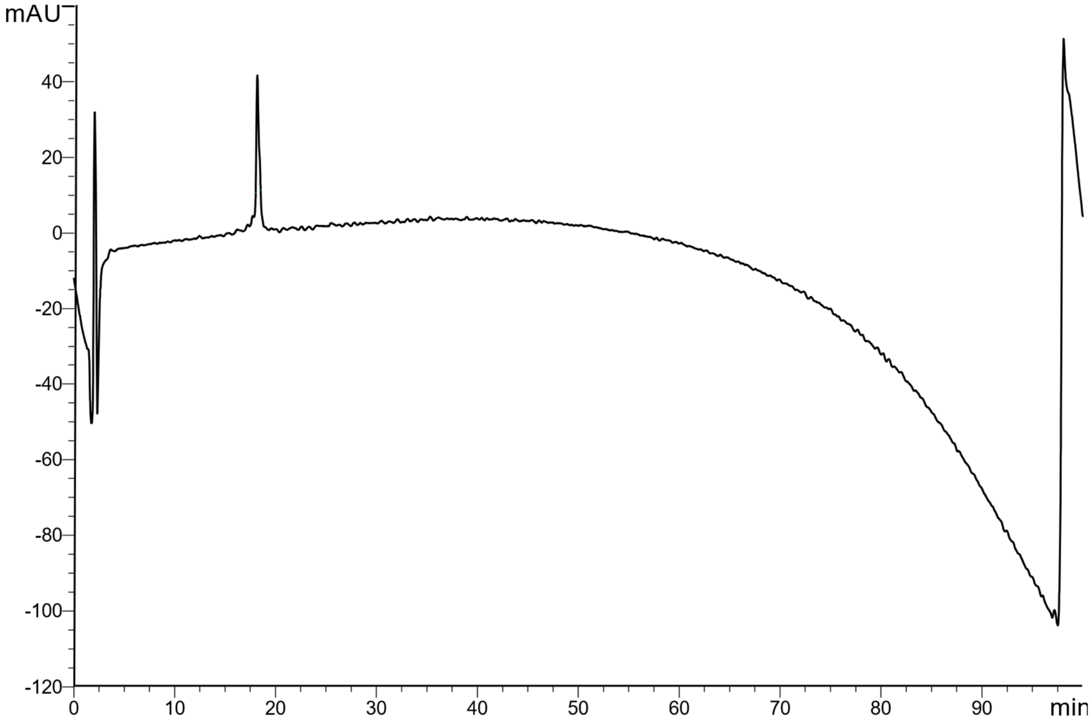

Figure SC7: Analytical RP-HPLC chromatogram of purified peptide **11**, t_R = 18.2 min. Chromatographic separations were performed on a Thermo Scientific Dionex Ultimate 3000 HPLC using a XTerra® MS C-18 column (5 µm; 4.6 × 150 mm) and a linear gradient of 5-95% B in 95 min at room temperature, *ca*. 1% B per min at a flow rate of 1.0 mL/min. Buffer A: H_2O containing 0.1% TFA (*v/v*); Buffer B: acetonitrile containing 0.1% TFA (*v/v*).

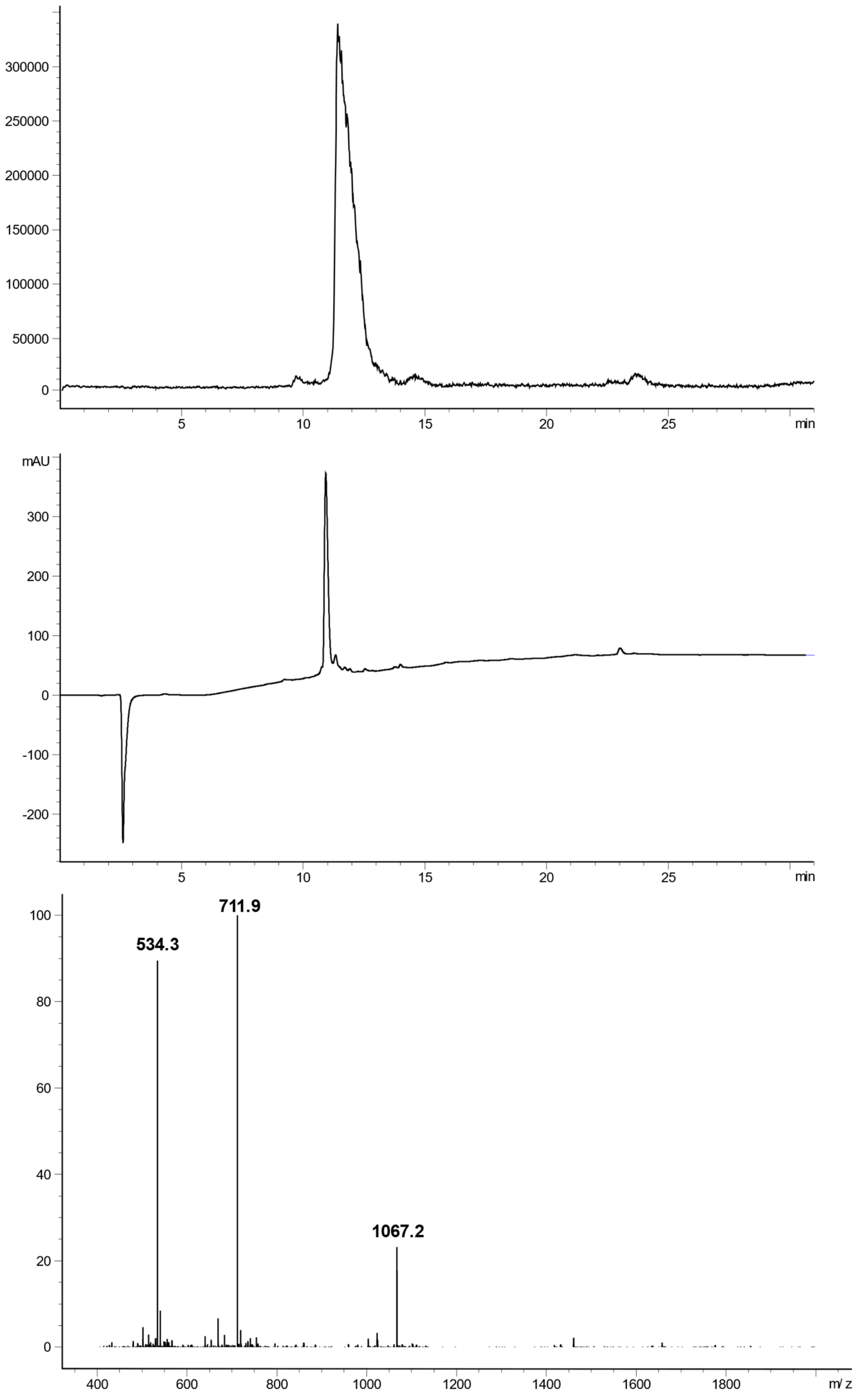

Figure SC8: LC-MS profile of purified peptide **11**; ion polarity positive operating at a nominal accelerating voltage of 70 eV. ESI-MS (*m/z* $[M+2H]^{2+}$ calcd: 1067.7; found: 1067.2; $[M+3H]^{3+}$ calcd: 712.1; found: 711.9; $[M+4H]^{4+}$ calcd: 534.4; found: 534.3. Mass deconvolution calculated at 2132.77 Da with a standard deviation of 0.40 Da; theoretical mass calculated at 2133.40 Da.
